# Large‐Diameter DNA‐Scaffolded Nanopores Enabled by Loosely Packed Peptides for Single‐Molecule Sensing

**DOI:** 10.1002/anie.3311099

**Published:** 2026-05-22

**Authors:** Zugui Peng, Daisuke Noshiro, Shiroh Futaki, Ryuji Kawano

**Affiliations:** ^1^ Department of Biotechnology and Life Science Tokyo University of Agriculture and Technology Koganei‐shi Tokyo Japan; ^2^ School of Engineering Institute of Science Tokyo Meguro‐ku Tokyo Japan; ^3^ Institute for Genetic Medicine Hokkaido University Sapporo Hokkaido Japan; ^4^ Graduate School of Pharmaceutical Sciences Kyoto University Kyoto Japan

**Keywords:** DNA structures, nanotechnology, peptides, protein design, self‐assembly, single‐molecule studies

## Abstract

Creating alternative nanopores is crucial for advancing single‐molecule methodologies. Peptide nanopores scaffolded by DNA nanostructures have emerged as a promising class of chemically synthesizable nanopores with organized geometries. However, no such nanopores have yet achieved single‐molecule detection due to size limitations. Here, we overcome this limitation by employing the alamethicin (ALM) peptide, based on the hypothesis that peptides with looser helix–helix packing are advantageous for constructing large‐diameter nanopores. The resulting DNA–ALM nanopores exhibited long‐lived open states and conductance values comparable to those of natural protein nanopores. To further enhance pore functionality, we introduced asymmetric electrostatic forces by harnessing the intrinsic helix dipole of ALM, which facilitated pore expansion and improved ion transport efficiency. This modification led to enhanced conductance levels and enabled single‐molecule sensing of biomolecules, including DNA and peptides. Overall, this work demonstrates a generalizable strategy for constructing functional, chemically synthesizable nanopores, providing a foundation for programmable nanopore design and next‐generation biosensing applications.

## Graphical abstract

A nanopore composed of transmembrane peptides and a DNA scaffold that assists peptide assembly was constructed. By employing peptides containing the unnatural amino acid 2‐aminoisobutyric acid (Aib), which promotes loose packing between helices, a large‐diameter nanopore was achieved. The resulting nanopores enable the translocation of single biopolymers, such as DNA and peptides, and allow their discrimination based on molecular weight.

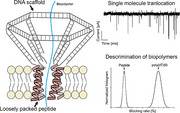

## 1 Introduction

Biological nanopores have expanded the possibilities for characterizing molecules at the single‐molecule level [1, 2, 3, 4, 5, 6, 7]. Starting from alamethicin (ALM) [8] and following α‐hemolysin (αHL) [9, 10], several transmembrane proteins have since been developed as ultrasensitive nanopore sensors, including *Mycobacterium smegmatis porin A* (MspA) [11, 12], fragaceatoxin C (FraC) [13, 14], aerolysin [15, 16], and the outer membrane lipoprotein CsgG [17]. Since the performance of nanopore technology strongly depends on both the physical (e.g., topology, rigidity) and chemical (e.g., hydrophobicity, surface charge) properties of the nanopore, each of the nanopores above exhibits its own strengths. Developing alternative classes of nanopores will further expand the capabilities of nanopore technology.

Over the last decade, the construction of nanopores other than native transmembrane proteins has become a reality. These include de novo proteins [18, 19, 20], peptides [21, 22, 23, 24, 25, 26], and DNA nanostructures [27, 28, 29, 30, 31]. Each class offers distinct advantages; however, from the perspective of developing nanopore sensors, peptide‐based nanopores currently appear to provide several practically useful features for the following reasons: (i) peptides can form nanopores with relatively large pore diameters suitable for analyte translocation, which remains a challenge for de novo protein‐based pores [18]; (ii) protein expression in *Escherichia coli* is often unpredictable and may result in low yields or impure products, whereas peptides can be reliably synthesized using solid‐phase peptide synthesis, allowing greater design flexibility; and (iii) the chemical environment of the inner lumen of peptide nanopores is more tunable than that of DNA nanopores, which typically present uniformly negatively charged surfaces. So far, several groups, including ours, have been working on constructing functional peptide nanopores formed from both α‐helical [26, 32, 33, 34] and β‐sheet [23, 35] secondary structures.

**FIGURE 1:**
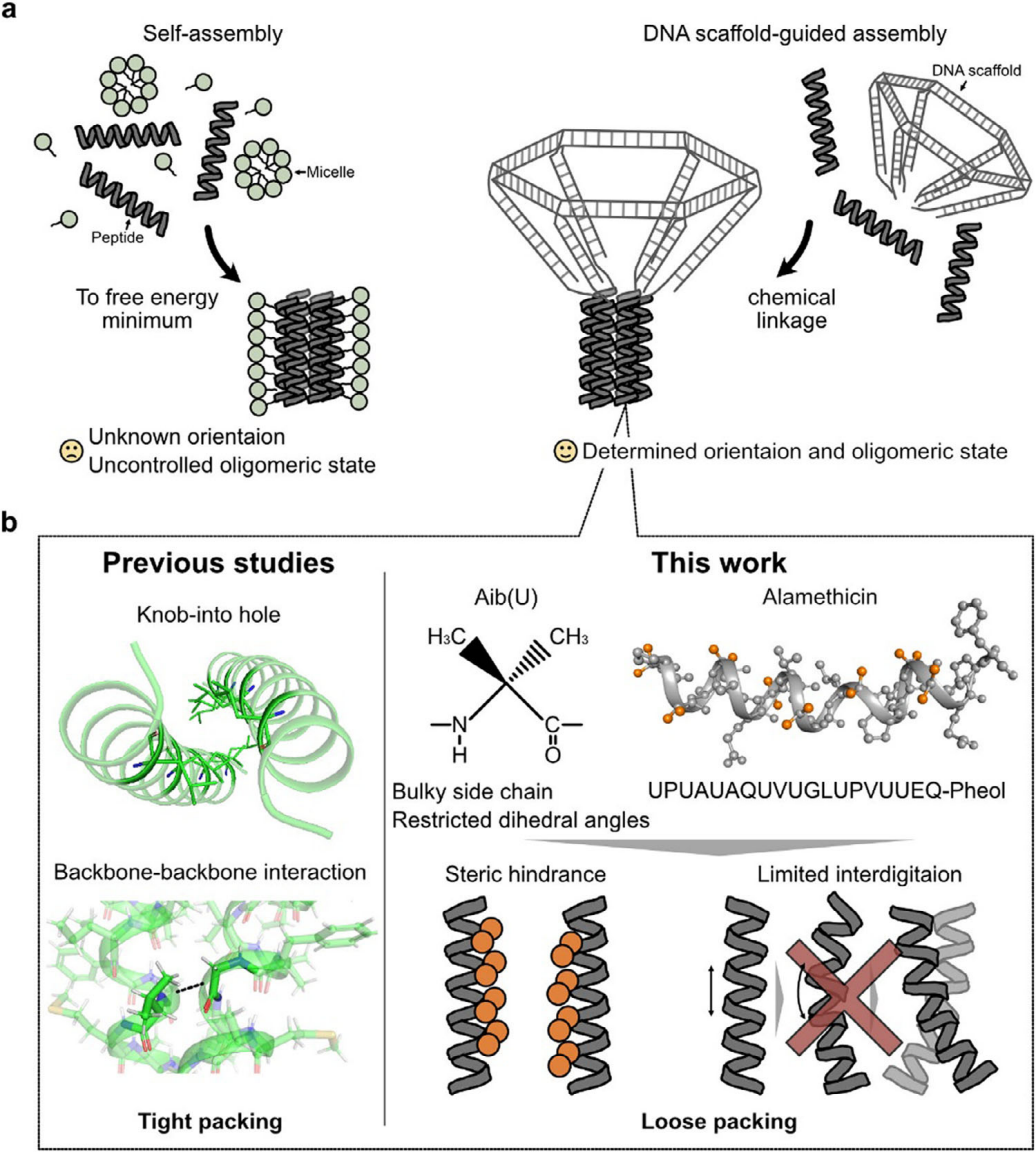
DNA‐scaffold‐guided assembly for nanopore formation. (a) Incubation with micelles can assist peptide assembly, but the resulting oligomeric number and peptide orientation are not controllable. Chemically linking the peptides to DNA scaffolds directs their oligomerization and enables the formation of nanopores with defined geometry. (b) Previous studies used peptides that pack tightly against each other, which limits the achievable pore diameter. In this work, alamethicin (ALM), a peptide with an Aib‐rich sequence, was incorporated into the DNA scaffold. The bulky side chains and restricted dihedral angles of Aib cause ALM peptides to pack only loosely with one another. Such looser packing allows the formation of DNA‐scaffolded nanopores with diameters large enough for single‐molecule detection.

A fundamental requirement for constructing large peptide pores is that free peptides pre‐assemble in aqueous solution before membrane insertion, thereby ensuring the formation of a single pore. One common strategy is to incubate peptides with micelles, where they aggregate into pore‐like structures within the micelle core [36]. This approach, however, offers little control over the oligomeric number or peptide orientation. Moreover, the assembled peptides often dissociate upon insertion into the lipid membrane, leading to nanopore instability. To overcome this limitation, researchers have employed DNA nanostructures as programmable scaffolds to guide the assembly of transmembrane proteins and peptides (Figure 1a) [37, 38, 39, 40]. Two studies, in particular, demonstrated that DNA scaffolds can direct the oligomerization of α‐helical Wza [38] and CtxA [39] peptides, resulting in stable pore structures. Such DNA‐scaffolded nanopores may further expand the design space of nanopore engineering [41], as both DNA and peptides are highly designable materials that can be obtained by chemical synthesis. However, in the study of scaffolded Wza pores, the authors did not demonstrate analyte translocation, nor did they discuss whether or why achieving translocation might be challenging. In the scaffolded ClyA pore study, the authors attempted single‐molecule translocation measurements but were unsuccessful. Therefore, to date, no molecular translocation has yet been observed in DNA‐scaffolded nanopores, suggesting that these systems still fall short of functioning as reliable nanopore sensors. One possible explanation is that the DNA scaffold itself may hinder analyte entry into the pore [39]; however, further investigation is required to clarify this point. On the other hand, we have also noticed that the peptides used so far in DNA‐scaffolded nanopore studies tend to pack relatively tightly with one another. For example, the Wza‐derived peptides pack through weak knobs‐into‐holes (KIH) interactions, which rely on the tight interdigitation of side chains [42], whereas CtxA appears to follow small‐residue packing motifs, in which small amino acids (G, A, or S) occur in small‐xxx‐small patterns, thereby enabling stabilizing interhelical backbone hydrogen‐bonding interactions [43]. Such tight packing is generally favorable for creating a stable pore, but, on the other hand, may produce pores that are too small for target‐molecule translocation.

To create a truly functional DNA‐scaffolded nanopore with a larger diameter, we focus on peptides that exhibit looser packing, namely peptides whose packing is not governed by either KIH interactions or small‐residue motifs (Figure 1b). ALM is a 20‐residue pore‐forming peptide produced by the fungus *Trichoderma viride*, with an α‐helical segment at the N‐terminus (residues 1–10) and a 3_10_‐helical segment at the C‐terminus (residues 12–20) [44]. It is well known that ALM inserts into lipid membranes in a voltage‐dependent manner, as the helix dipole facilitates insertion from the N‐terminus when a negative voltage is applied to the trans side of the membrane [45], and that this peptide packs in lipid bilayers and forms nanopores with multiple conductance levels through the successive association and dissociation of ALM monomers [46, 47, 48, 49, 50, 51]. The packing is primarily mediated by hydrophobic interactions involving the side chains of the unnatural amino acid 2‐aminoisobutyric acid (Aib) [52]. The two bulky methyl groups of Aib create a shielded helix surface, resulting in steric hindrance that prevents helices from approaching each other too closely [53]. Moreover, because the methyl groups are attached to the α‐carbon, the side chains are too short to form a KIH packing arrangement. Another characteristic of Aib is its restricted dihedral angles [54], which make it a strong helix former and produce a relatively rigid helix with limited bending flexibility. As a result, ALM helices do not readily interdigitate, making it difficult to optimize local helix–helix contacts to form a typical α‐barrel. Instead, ALM nanopores are frequently described as “barrel‐stave” structures, in which the helices stand nearly perpendicular to the lipid membrane [55]. Therefore, compared with other pore‐forming peptides, ALM exhibits looser packing and can lead to a larger pore diameter. This is evident from the fact that, for example, an octameric cWza nanopore exhibits a conductance of approximately 1 nS in 1 M KCl solution [25], whereas an octameric ALM nanopore exhibits a conductance of about 4 nS under the same conditions [46].

Here, we use the loosely‐packed ALM to construct the first DNA‐scaffolded functional nanopores, combining long‐term stability with a large pore diameter. We show that DNA‐scaffolded ALM peptides preferentially assemble into hexameric nanopores, while increasing the number of ALM monomers leads to unstable assemblies and rarely produces stable pores with conductance matching that of their unscaffolded counterparts. Although the hexameric DNA‐ALM nanopores exhibit conductance comparable to that of αHL, we did not observe any molecular translocation in their original form. To address this, we modified the attachment site of the DNA on ALM to exploit the helix dipole, thereby introducing an opposing force acting on the peptides. This force imbalance facilitates the formation of nanopores with larger conductance, suggesting expansion of the pore diameter. Finally, we confirmed the sensing capability of the DNA‐ALM nanopores and found that they can discriminate between biomolecules, including DNA and peptides.

## 2 Results and Discussion

### 2.1 An Engineered Covalent Dimer of ALM Provides Evidence for the Potential of ALM Nanopores as Single‐Molecule Sensors

**FIGURE 2:**
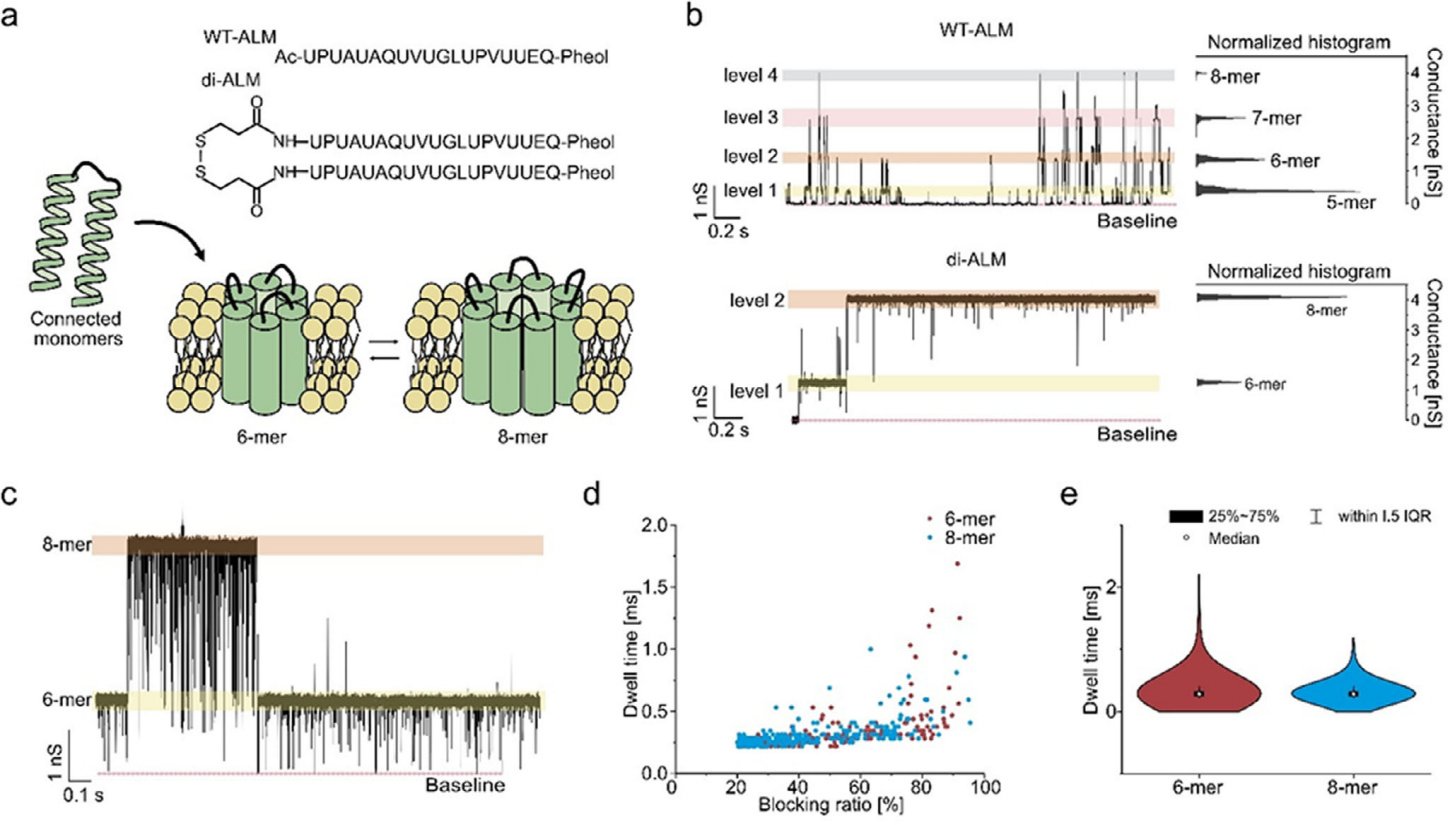
Demonstration of the potential of ALM nanopores as a single‐molecule sensor. (a) Amino acid sequences of wild‐type ALM (WT‐ALM) and di‐ALM, together with a schematic illustration of di‐ALM pore formation in lipid membranes. (b) Representative current traces and conductance histograms of WT‐ALM and di‐ALM nanopores. Recordings were performed in 1 M KCl, 10 mM MOPS buffer (pH 7.0) at applied voltages of +150 mV (WT‐ALM) and +100 mV (di‐ALM). Conductance levels corresponding to distinct oligomeric states are highlighted. (c) Representative current traces of di‐ALM nanopores in the presence of 1 µM poly(dA)_50_ added to the *cis* chamber in 1 M KCl, 10 mM MOPS buffer (pH 7.0), at an applied voltage of +120 mV. (d) Scatter plots of dwell time versus blocking conductance for poly(dA)_50_ translocation events through hexameric and octameric di‐ALM nanopores. (e) Violin plots of dwell times for poly(dA)_50_ translocation events through hexameric and octameric di‐ALM nanopores.

As the wild‐type ALM (WT‐ALM) is known to form nanopores composed of 4–11 monomers [56], we first sought to determine which oligomeric states are suitable for single‐molecule sensing. However, direct use of WT‐ALM is extremely challenging because of its very short lifetime. To this end, we investigated the sensing ability of previously reported N‐terminally linked ALM dimers (di‐ALM) [46], which exhibit longer lifetimes and assemble only into hexameric or octameric states (Figure 2a and Table S1). The di‐ALM was synthesized by solid‐phase peptide synthesis and purified by reversed‐phase high‐performance liquid chromatography (RP‐HPLC). The pore‐forming ability of di‐ALM was confirmed by electrical recordings using 1,2‐diphytanoyl‐sn‐glycero‐3‐phosphocholine (DPhPC) bilayers prepared by the droplet contact method on a microdevice with two chambers in 1 M KCl, 10 mM MOPS (pH 7.0) solution (Figure S1) [57]. Peptides were added to the *trans* side of the chamber. Figure 2b shows representative current traces for WT‐ALM and di‐ALM. Both peptides exhibited multiple conductance open levels, suggesting dynamic association and dissociation of peptide monomers within the nanopores as a result of peptide insertion into and dissociation from the lipid membrane. However, the lifetimes of these states were different. WT‐ALM pores had an average lifetime of 1.1 ± 0.8 ms, whereas di‐ALM pores exhibited a substantially longer average lifetime of 639.4 ± 926.8 ms (*n* = 50) (Figure S2). Conductance histograms revealed four open levels for WT‐ALM, with peaks ranging from 0.4 nS to 4 nS, corresponding to pentameric to octameric pores. In contrast, di‐ALM displayed only two peaks at 1.3 and 4.1 nS, which are consistent with the formation of hexameric and octameric pores (see also Table S2 for the estimated pore diameters). Additionally, we observed that di‐ALM formed pores more readily than WT‐ALM, as pore formation was initiated at +100 mV, whereas WT‐ALM required potentials greater than +150 mV (Figures S3 and S4). This difference may be attributed to locally concentrated ALM molecules forming pores with a lower energy barrier [50].

The sensing ability of di‐ALM was then examined by using it to detect single‐stranded poly(dA)_50_ oligonucleotides. 1 mM poly(dA)_50_ was added to the *cis* side of the chamber. Potentials were applied to electrophoretically drive the poly(dA)_50_ into the pores. Distinct current‐blocking signals, which reflect ion blockade through the pores caused by poly(dA)_50_ translocation, were observed in both hexameric and octameric pores (Figure 2c). The scatter plots of the blocking signals from the two pore types showed substantial overlap and thus did not provide a sufficiently clear distinction in their sensitivity (Figure 2d). Although no significant difference in dwell time was detected between the two pore types using the Mann–Whitney *U*‐test, violin plot analysis revealed that signals from hexameric pores were more frequently distributed at longer dwell times than those from octameric pores, with dwell‐time ratios ≥ 8 ms of 4.4% and 1.6%, among all signals, respectively (Figure 2e). As longer dwell times indicate that the sensing target remains within the pore lumen for a greater duration and thus yield more information about the target molecules, hexameric pores appear to be more beneficial for nanopore sensing at this stage. Therefore, we shifted to constructing a hexameric ALM nanopore by using DNA nanostructures as a scaffold.

### 2.2 A DNA Scaffold Can Support the Assembly of ALM Monomers into a Stable Hexameric Nanopore

**FIGURE 3:**
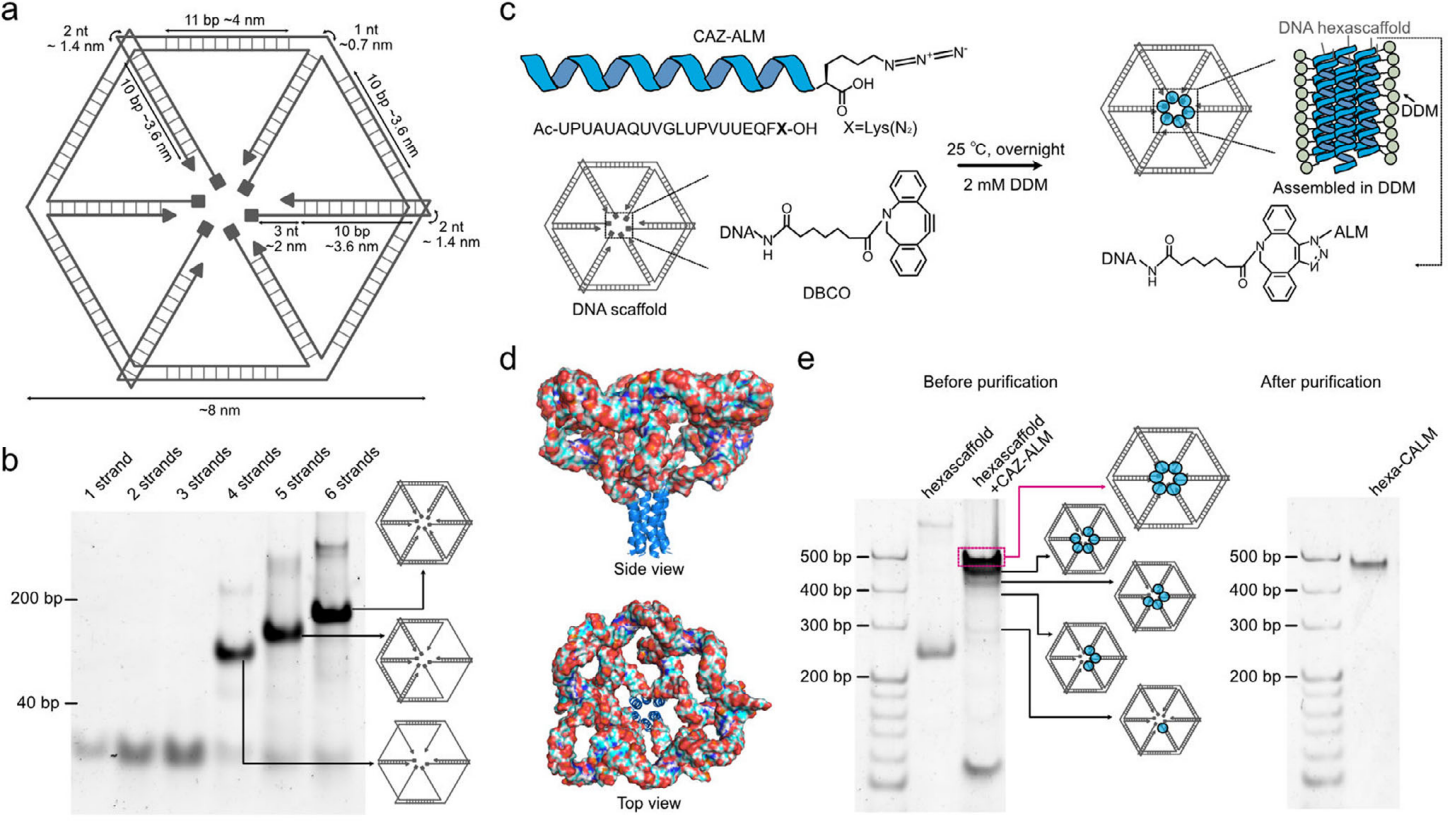
Design and synthesis of the hexa‐CALM nanopore. (a) Two‐dimensional schematic of the six‐armed DNA scaffold (hexascaffold), composed of six single DNA strands. (b) 5% Polyacrylamide gel electrophoresis (PAGE) of assemblies formed from one to six component strands of the hexascaffold (lanes 1–6). (c) Schematic representation of the reaction between the ALM peptide containing *N* ^6^‐diazo‐l‐lysine (Lys(N_2_)) at the C terminus (CAZ‐ALM) and the pre‐annealed hexascaffold. The hexascaffold was modified at the 5′ terminus with dibenzocyclooctyne (DBCO). Peptides are directly clicked onto the pre‐assembled DNA scaffold (assembly‐first, conjugation‐later approach). (d) Structural model of the hexascaffold conjugated with six CAZ‐ALM peptides. (e) 5% PAGE of the reaction mixture of CAZ‐ALM and the hexascaffold (left), and of the purified, fully conjugated hexa‐CALM.

We designed a ring‐shaped DNA scaffold with six arms (hexascaffold) to support the assembly of six ALM monomers into a hexameric pore (Figure 3a; Tables S3 and S4). This design was simplified from the DNA scaffold used for the assembly of 12 Wza monomers reported by Spruijt et al. [38]. The DNA scaffold consists of six single DNA strands, each comprising two segments that hybridize to form the ring and two additional segments that extend as arms. Unhybridized bases were introduced between each segment to provide structural flexibility. The ALM peptide was designed to attach to the 5′ terminus of each DNA strand, with an additional three unhybridized nucleotides placed at the 5′ terminus to separate the DNA from the peptide. The hexascaffold was prepared in 10 mM Tris, 1 mM EDTA, and 20 mM MgCl_2_, pH 8.0 (TE20). Formation of the hexascaffold was supported by gel electrophoresis, which showed a single band migrating to a higher position as more DNA components were included (Figure 3b).

The ALM peptide with *N* ^6^‐diazo‐l‐lysine (Lys(N_2_)) at the C terminus (CAZ‐ALM) was attached to the dibenzocyclooctyne (DBCO)‐modified 5′ terminus of the pre‐annealed hexascaffold through a strain‐promoted alkyne–azide cycloaddition (SPAAC) reaction in TE20 buffer at 25°C overnight (Figure 3c). To solubilize the peptides (Figure 3d), 2 mM *n*‐dodecyl‐β‐d‐maltoside (DDM) was included. The attachment of CAZ‐ALM to a single DNA strand, with an overall yield of more than 95%, was confirmed by HPLC (Figure S5). Since each hexascaffold contains six DBCO groups and the reaction efficiency was less than 100%, we observed six distinct bands corresponding to hexascaffolds conjugated with different numbers of CAZ‐ALM monomers (Figure 3e). We cut out the bands corresponding to fully conjugated hexascaffold (hexa‐CALM) and purified them by soaking the gel in TE20 buffer containing 2 mM DDM. Successful purification was confirmed by the presence of a single band in the polyacrylamide gel of the extract. To further verify the purity of hexa‐CALM, we adopted a conjugation‐first, assembly‐later approach, which removes unreacted monomers during the initial denaturing PAGE purification (Figure S6). The molecular weights of the scaffolds containing different numbers of peptides match well with those obtained using the assembly‐first, conjugation‐later approach (Figure 3e), indicating that free CALM monomers did not co‐migrate with the final scaffolded peptide assemblies and that micelle‐associated clusters are absent. As the assembly‐first, conjugation‐later approach is easier to perform, all subsequent experiments were conducted using this method.

**FIGURE 4:**
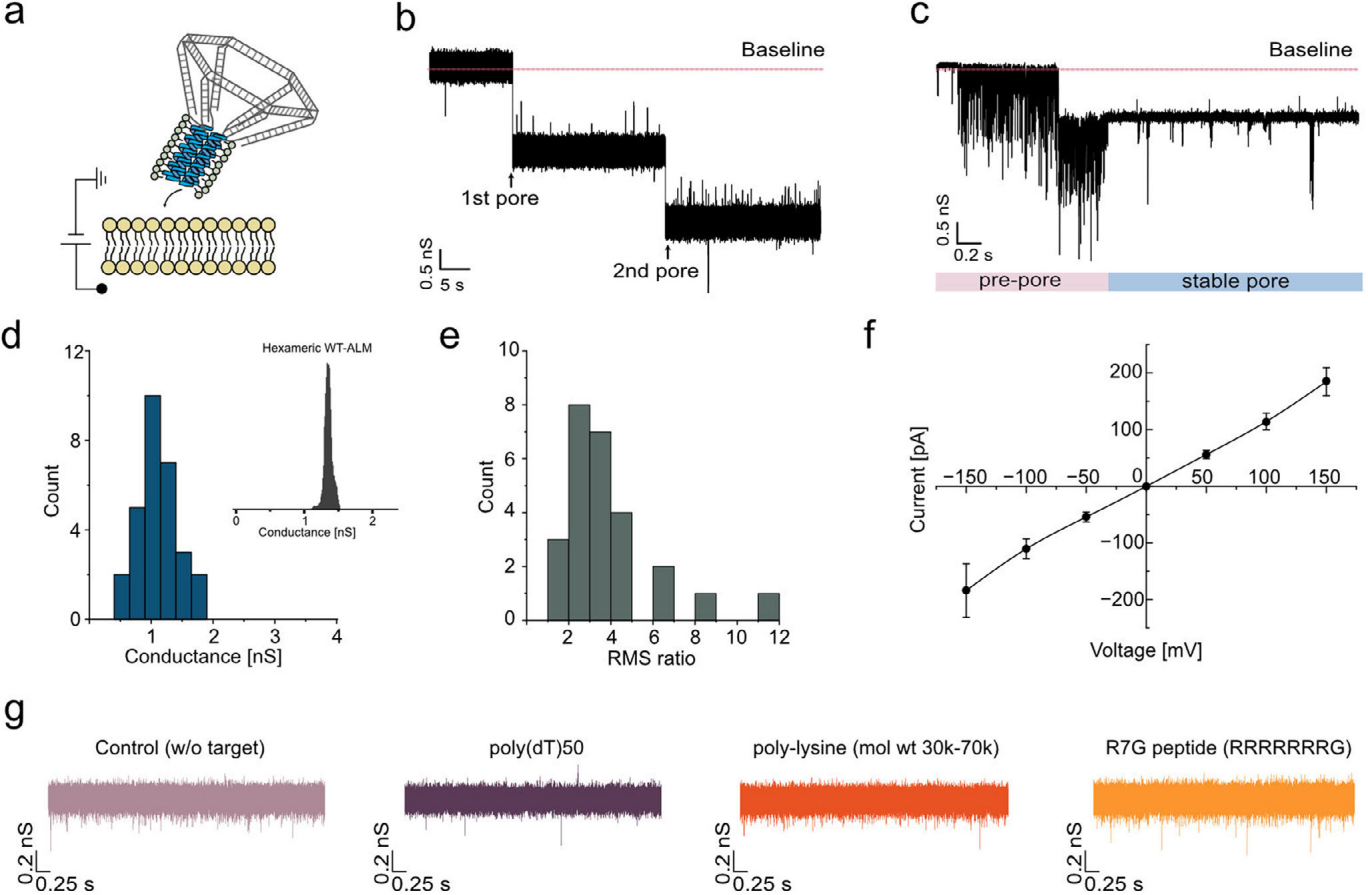
Electrical characterization of the hexa‐CALM nanopores. (a) Schematic illustration of the electrical recording experiment. (b and c) Representative current traces of hexa‐CALM nanopores recorded at applied potentials of (b) −100 mV and (c) −200 mV. (d and e) Histograms of (d) conductance values and (e) RMS ratio at −100 mV (*n* = 26). The inset in (d) shows the conductance histogram of hexameric WT‐ALM, corresponding to the same data presented in Figure 2b. (f) Average *I*–*V* curve obtained from five individual hexa‐CALM nanopores. Error bars represent standard deviations. (g) Representative current traces of hexa‐CALM nanopores in the absence and presence of poly(dT_50_), poly‐l‐lysine (PLL), and the R7G peptide. Concentrations of each sensing target ranged from 5 µM to 50 µM.

The pore‐forming ability of hexa‐CALM was confirmed by electrical recordings using DPhPC bilayers in 1 M KCl, 10 mM MOPS (pH 7.0) solution (Figure 4a). The extract was diluted 25‐fold into the *cis* chamber, resulting in a final DDM concentration of 80 µM. This DDM concentration was confirmed to have little effect on the recording signals in the absence of hexa‐CALM (Figure S7). Spontaneous pore insertion was observed as multiple‐step current signals at an applied potential of −100 mV (Figure 4b), indicating an organized assembly of the ALM inside the lipid membrane compared with the unscaffolded CAZ‐ALM (Figure S8). Pore insertion was further enhanced by increasing the voltage to −200 mV, leading to erratic recording signals corresponding to pre‐pore insertion, followed by a stable intermediate conductance level indicative of stable pore formation (Figure 4c). This behavior is likely due to higher voltages facilitating the insertion of ALM from its N terminus as also observed in WT‐ALM [45]. Considering that translocation of the highly negatively charged DNA scaffold is energetically unfavorable, we infer that the N terminus of hexa‐CALM inserts into the lipid membrane first.

The pores exhibited a mean unitary conductance of 1.1 ± 0.3 nS, which is comparable to that of hexameric WT‐ALM nanopores (Figure 4d). Approximately 85% of the single pores showed a root‐mean‐square (RMS) noise‐to‐baseline ratio below 4 at −100 mV, further demonstrating their stability (Figure 4e). Current–voltage (*I*–*V*) characterization showed that the conductance of hexa‐CALM nanopores varied approximately linearly with the applied voltage, with *−I*_−__50_/*I*_+__50_ = 1.03, indicating negligible rectification (Figure 4f). The pores were stable, and no gating events were observed over 1 h at applied potentials between −100 mV and +100 mV (Figure S9). Taken together, the unitary conductance, high stability, negligible rectification, and absence of gating render hexa‐CALM comparable to the αHL nanopore, which is widely used for nanopore sensing [58].

Although the pore characterization data support the potential of hexa‐CALM for nanopore sensing, we were unable to observe single‐molecule translocation events through these pores. Single‐molecule sensing experiments were performed at an applied potential of +100 mV, with target molecules added to the chamber where electrophoretic force was expected to drive their translocation through the pore. Specifically, negatively charged poly(dT)_50_ oligonucleotides were added to the *cis* chamber, whereas positively charged poly‐l‐lysine (PLL, Mw = 30 000–70 000) and the R7G peptide (RRRRRRRG) were added to the *trans* chamber. We selected poly(dT)_50_ instead of poly(dA)_50_, which was used in the di‐ALM sensing tests, because poly(dT)_50_ behaves more like a flexible random polymer than poly(dA)_50_ [59, 60]. No distinguishable blocking signals from the sensing targets were observed (Figure 4g). Because the pores tended to become unstable at voltages higher than +100 mV, we did not perform experiments at higher applied potentials. However, we do not attribute the lack of translocation events to an insufficient driving force, since the translocation of DNA, PLL, and R7G peptides has been confirmed at +100 mV in previous studies [61, 62, 63]. Similar non‐ideal translocation behavior has also been reported for the octameric DNA‐scaffolded CtxA nanopore, where the authors suggested that the DNA scaffold impeded molecular entry into the pore [39]. In our case, however, it remains unclear whether a similar phenomenon occurs in hexa‐CALM nanopores, as we could not detect translocation of targets such as R7G regardless of whether they were added to the *cis* or *trans* chamber (Figure S10). Another possible explanation is that the negatively charged residue E17 may hinder the entry of negatively charged DNA, although such an effect would be expected to promote, rather than inhibit, the translocation of positively charged molecules such as PLL and R7G [64]. Using the Hille equation, the theoretical diameter of the 1.1 nS hexa‐CALM nanopore is estimated to be 0.77 nm. This value is smaller than the effective diameter of DNA (∼1 nm) and is also comparable to or smaller than that of peptides (∼0.5–0.8 nm) [65], considering the uncertainty in the estimation. Taken together, these results lead us to consider that the small pore size may be responsible for the lack of molecular entry.

### 2.3 Enlarging the Pore Size by Scaffolding a Larger Number of ALM Monomers is Challenging

**FIGURE 5:**
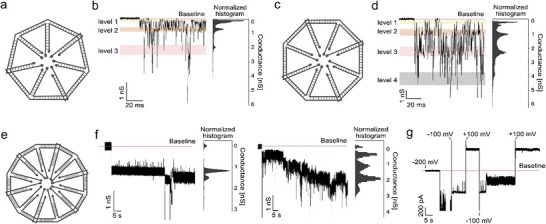
Design, synthesis, and electrical characterization of hepta‐, octa‐, and dodeca‐CALM nanopores. (a) Two‐dimensional schematic of the seven‐armed DNA scaffold (heptascaffold), composed of seven single DNA strands. (b) Representative current traces of hepta‐CALM nanopores recorded at an applied potential of −200 mV. (c) Two‐dimensional schematic of the eight‐armed DNA scaffold (octascaffold), composed of eight single DNA strands. (d) Representative current traces of octa‐CALM nanopores recorded at an applied potential of −200 mV. (e) Two‐dimensional schematic of the 12‐armed DNA scaffold (dodecascaffold), composed of 12 single DNA strands. (f) Representative current traces showing a spontaneous step‐like increase in conductance of dodeca‐CALM nanopores at an applied potential of −100 mV. (g) Representative trace of a dodeca‐CALM nanopore that appeared erratic at −200 mV but stabilized at −100 mV.

To create pores with larger diameters, we assembled a greater number of CAZ‐ALM peptides using DNA scaffolds with additional arms to accommodate more subunits. However, the majority of nanopores with seven or eight peptides (hepta‐CALM and octa‐CALM) exhibited unstable, multiple conductance levels, reminiscent of the behavior of unscaffolded CAZ‐ALM, although the lifetime of each conductance state appeared to be even shorter (Figures 5a–d and S11a,b). The conductance peaks for hepta‐CALM were 0.7 and 2 nS, whereas those for octa‐CALM were 0.9, 2.2, and 3.8 nS. These values are comparable to the conductance values of 1.3, 2.6, and 4 nS for hexameric to octameric WT‐ALM. In rare cases, both hepta‐CALM and octa‐CALM produced single, step‐like signals indicative of individual pore insertions, but these pores also showed a wide variation in conductance (1–4 nS) and consistently exhibited poor baselines or short lifetimes (Figures S12 and S13). Combined with the previously demonstrated stable pore formation by hexa‐CALM, our results suggest that ALM preferentially assembles into a hexameric structure, whereas the addition of one or two extra peptides destabilizes the assembly rather than supporting the formation of a pore with a larger diameter. This is because, in order to form a pore structure, the peptides are constrained to adopt a defined oligomeric state with a specific helix–helix packing geometry. In this arrangement, hydrophobic residues must face the lipid core or interact with one another, while hydrophilic regions are oriented toward the pore lumen. Altering the oligomeric number forces rotation and rearrangement of the peptides, disrupting this optimal packing. Such rearrangements may expose hydrophobic residues to the aqueous environment, which is energetically unfavorable and can ultimately lead to pore instability or collapse. Similar results have been reported in a previous study, where one or two peptides were observed to be excluded from heptameric and octameric WT‐ALM nanopore bundles, resulting in the formation of hexameric nanopores in MD simulations [66]. It should be noted that di‐ALM indeed forms a stable octameric nanopore. We attribute this difference to the translational and rotational freedom of the ALM monomers. In di‐ALM, the two ALM monomers are constrained by a short linker (>10 Å), which is shorter than half of the length of the linker between ALM and DNA in the DNA‐scaffolded ALM system. As a result, each ALM monomer in di‐ALM experiences stronger local restraints and may therefore tolerate energetically unfavorable conformations. However, the detailed mechanism remains to be elucidated.

Although our results indicate that ALM favors hexameric assembly, we next attempted to scaffold dodecameric ALM using DNA (dodeca‐CALM), an oligomeric state not found in nature (Figures 5e and S11c). Interestingly, while multilevel conductance or step‐like single‐pore insertions with lifetimes shorter than 5 s accounted for 47% of the recordings (7 of 15 independent experiments), signals showing a spontaneous, step‐like increase in conductance were observed in 33% of the cases (5 of 15 independent experiments), likely indicating the association of additional peptides after pore insertion (Figures 5f and S14). The remaining two cases initially appeared as large and erratic pores at −200 mV but became stable when the applied potential was decreased to −100 mV (Figures 5g and S15). Both pores exhibited conductance values of 5–6 nS, which are comparable to those of a single nonameric WT‐ALM nanopore and greater than the combined conductance of two hexameric WT‐ALM nanopores. Therefore, we considered them to represent a single nanopore, but the actual oligomeric state should be further verified. The *I*–*V* curve of the nanopore shown in Figure 5g was approximately linear with the applied voltage, with *−I*_−__50_/*I*_+__50_ = 0.99, which is comparable to that of the hexa‐CALM nanopore (Figure S16). The gating of pores could be attributed to voltage‐triggered conformational changes of the pore [67]. We then confirmed the sensing ability of the pore using R7G. After adding 40 µM R7G peptides to the trans side of the chamber, clear blocking signals arising from interactions between the nanopore and R7G were observed (Figure S17). The blocking signals may reflect translocation of R7G through the pore; however, further analysis is required (Supporting Information: Explanation 1). In summary, expanding the pore size by increasing the number of scaffolded ALM peptides may offer some potential, but its feasibility appears limited. Because the low pore formation efficiency and poor experimental reproducibility made the experiments challenging, we did not further investigate their sensing abilities.

### 2.4 Harnessing the Helix Dipole to Tune Helix Assembly for the Formation of Large‐Diameter Pores

Tuning the pore diameter using DNA scaffolds is a strategy that has been demonstrated in previous studies [37, 38, 39], and our results show partial agreement with these findings, indicating that the ALM peptide adopts a defined assembly number for stable nanopore formation. On the other hand, we additionally found that the voltage‐dependent insertion of ALM, driven by their helix dipole, persists even after DNA conjugation and still strongly influences the pore formation process of DNA‐scaffolded ALM nanopores, as shown in Figures 3c and 5g. Apparently, the insertion of ALM is a dynamic process involving peptide tilting during membrane insertion, and it relies on a fragile balance, as even small fluctuations in applied voltage can markedly affect channel formation [51]. Meanwhile, the first report on α‐helical peptide nanopores has highlighted that the pore size can vary depending on the tilt angle of the helices relative to the lipid membrane normal [25]. Considering our present findings together with earlier reports and the inherently weak helix–helix interactions of ALM, we hypothesized that harnessing the helix dipole to modulate the tilt angle of the helices could provide a means to expand the pore size of DNA‐scaffolded nanopores.

**FIGURE 6:**
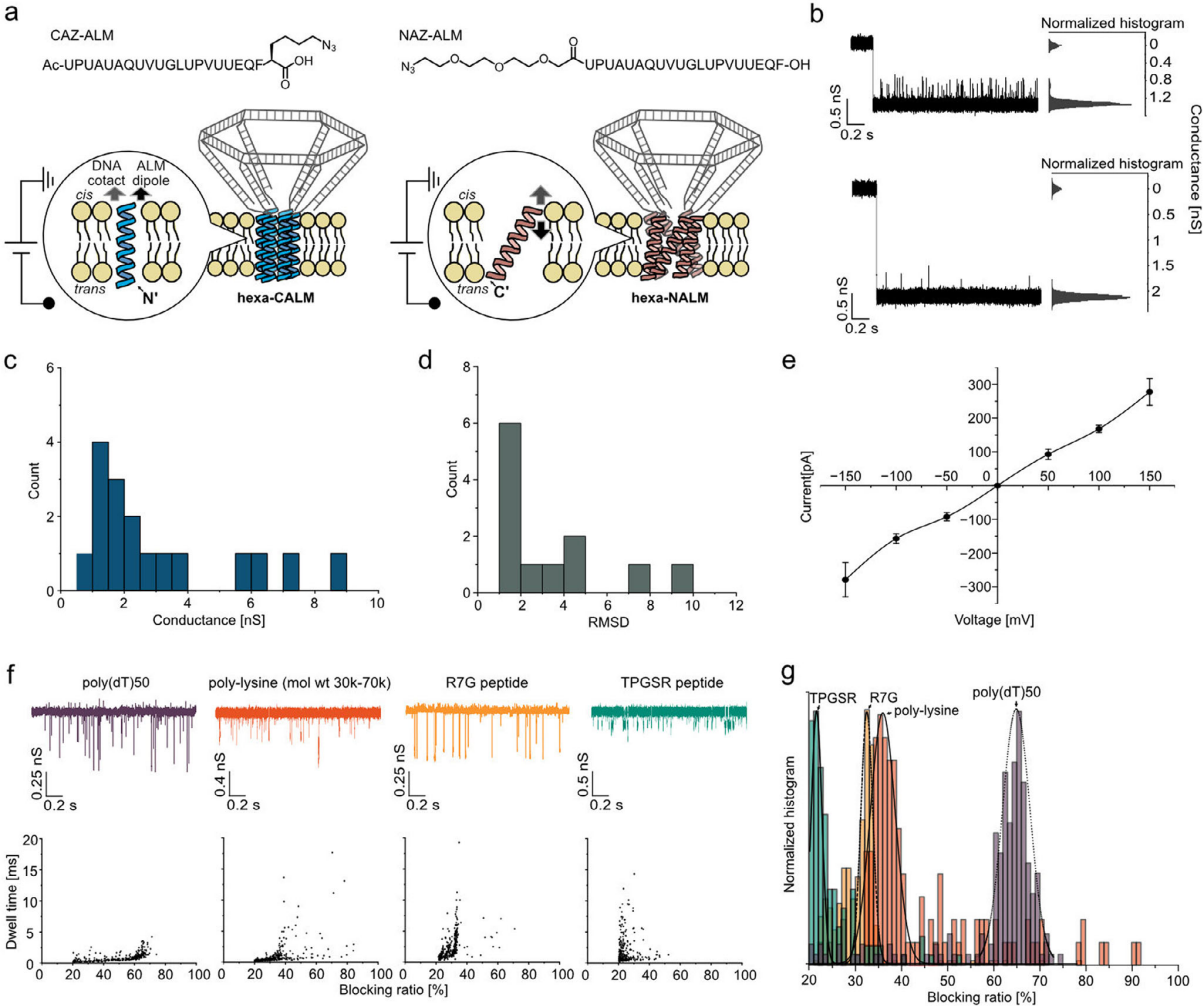
Tuning pore size by harnessing the helix dipole. (a) Schematic illustration of the external forces acting at the *cis* terminus of hexa‐CALM and hexa‐NALM nanopores. The black arrow indicates the electrostatic force derived from the helix dipole, and the gray arrow indicates the contact force that compensates for the electrostatic force acting on the DNA scaffold. (b) Representative step‐like current signals of hexa‐NALM nanopores at −100 mV. (c and d) Histograms of (c) conductance values (*n* = 17) and (d) RMS ratio at −100 mV (*n* = 12). (e) Average *I*–*V* curve obtained from three individual hexa‐NALM nanopores. Error bars represent standard deviations. (f) Representative current traces and scatter plots of blocking events for hexa‐NALM nanopores in the absence and presence of poly(dT)_50_, poly‐l‐lysine (PLL), R7G, and the TPGSR peptide. Concentrations of the sensing targets ranged from 100 nM to 2 µM. (g) Normalized histograms of the blockade ratio for events with dwell times longer than 1 ms, fitted with Gaussian functions.

We designed and synthesized an ALM analog with an 11‐azido‐3,6,9‐trioxaundecyl group at its N‐terminus (NAZ‐ALM), and performed a SPAAC reaction between this peptide and the hexascaffold. The resulting conjugates were purified by PAGE to obtain a alternative class of nanopores (hexa‐NALM) (Figures 6a and S18). We hypothesized that the NAZ‐ALM design may lead to a larger pore size for the following reasons: (i) the pore may experience a helix dipole‐derived electrostatic force [68] in the direction opposite to that acting on the DNA scaffold. This imbalance may induce tilting of the ALM helices, thereby contributing to pore enlargement (Supporting Information: Explanation 2). (ii) the PEG3 linker of the 11‐azido‐3,6,9‐trioxaundecyl group is more flexible than that of Lys(N_2_), which may further facilitate peptide tilting.

Obtaining clear step‐like signals corresponding to pore insertion was considerably more difficult than with hexa‐CALM, as ∼80% of the 125 independent experiments displayed only bursts and spike‐like events (Figure S19). This trend was consistent at both −100 mV and −200 mV, indicating that hexa‐NALM did not show voltage‐dependent insertion behavior. In the remaining ∼20% of cases, however, two distinct types of step‐like signals were observed (Figure 6b). The first type consisted of pores with conductance values around 1 nS, similar to those of hexa‐CALM, but with more frequent gating (Figure S20). More importantly, the second type consisted of pores with conductance exceeding 2 nS, with a broad distribution extending up to 8.7 nS, suggesting that these pores were heterogeneous rather than homogeneous (Figure 6c). These larger pores also exhibited gating, but unlike the first type, where currents increased and decreased reversibly, their current dropped unilaterally and returned to the open level only when the applied potential was reversed (Figure S21). Both pore types were noiseless, with 83% of the single pores showing an RMS noise‐to‐baseline ratio less than 4 (Figure 6d). Further evaluation of the *I*–*V* curves of the larger pores yielded a *−I*_−__50_/*I*_+__50_ value of 1.01 (Figure 6e).

The sensing ability of hexa‐NALM pores with conductance values between 2 nS and 3 nS was confirmed using the same protocol as that applied to hexa‐CALM. Spike‐like blocking events induced by poly(dT)_50_ oligonucleotides, PLL, R7G, and TPGSR, a small peptide containing only one charged residue [69], were observed under an applied potential of 100 mV (Figure 6f). Because short‐duration events are more likely to correspond to molecular collisions rather than translocations [70], the longer average dwell time of blocking events from R7G (2.7 ± 2.3 ms) and TPGSR (2.2 ± 2.9 ms) compared with poly(dT)_50_ (1 ± 0.7 ms) and PLL (2.1 ± 2 ms) suggests that the small R7G and TPGSR molecules undergo translocation more frequently than collision (mean ± standard deviation), in contrast to the longer oligonucleotides or polypeptides (Figures S22 and S23). The normalized histogram of the blocking ratio, defined as the fractional reduction in conductance upon analyte interaction with the nanopore, exhibited distinct peaks at 65%, 37%, 33%, and 22% for poly(dT)_50_, PLL, R7G, and TPGSR, respectively, based on events with durations longer than 1 ms (Figure 6g). The differences in blocking ratio likely arise from variations in the molecular mass of the sensing targets, as the values for all analytes fall within the expected ranges (Supporting Information: Explanation 1) [71, 72], and the plot of blocking ratio versus molecular weight shows a good linear correlation (Figure S24), consistent with previous results on peptide sensing using aerolysin nanopore [63]. Furthermore, the dwell time of spike signals with durations >1 ms during PLL detection decreases with increasing voltage (Figure S25), which is consistent with the behavior typically observed for molecular translocation events. This observation further suggests that the spike signals with durations >1 ms correspond to PLL translocation. Transport of molecules toward and through the pore is generally governed by a combination of forces, including electrophoresis, electroosmosis, and dielectrophoresis [73]. The ALM peptide is nearly neutral, therefore, no strong electroosmotic flow driven by pore lumen surface is expected. Although the negatively charged DNA scaffold could, in principle, induce cation selectivity and generate electroosmotic flow, this effect is only significant when the DNA is located very close to the pore entrance (i.e., at distances smaller than the Debye length) [74]. Because the linker between the DNA scaffold and the peptide has a length of approximately 3 nm, the DNA is expected to be too distant from the pore entrance to induce a relevant electroosmotic flow. Consequently, electrophoresis is expected to be the dominant transport mechanism for charged molecules. This is further supported by the nearly linear and symmetric *I*–*V* characteristics in Figure 6e, which suggest the absence of pronounced ion selectivity, and by the increased frequency of PLL translocation events at higher applied voltages (Figure S26). In addition, we performed the same TPGSR detection using di‐ALM nanopores but did not observe any apparent blocking signals, in contrast to the results obtained with hexa‐NALM nanopores (Figure S27). This is likely because the lifetime of di‐ALM pores is too short to capture TPGSR blocking events, given the approximate pore entry frequency of ∼5 events per second at a TPGSR concentration of 200 nM and an applied potential of 100 mV, as observed in scaffolded ALM pores. Taken together, these observations suggest that hexa‐NALM nanopores with conductance values greater than 2 nS are substantially more advantageous than non‐scaffolded ALM nanopores, as they are capable of supporting molecular translocation and, to some extent, discriminating single molecules based on differences in molecular weight.

## 3 Conclusion

Exploring and creating alternative nanopores with unique structures could open opportunities for developing nanotechnology applications such as biosensors, sequencers, and nanoreactors [11, 34, 75, 76, 77, 78]. In this study, we described a strategy that leverages ALM peptides with loose packing to form DNA‐scaffolded nanopores. These pores were able to insert into lipid membranes and maintained stable open‐pore conductance values for more than 1 h, which, to the best of our knowledge, has not been realized by previous approaches that tune ALM assembly using extramembrane peptide domains [47, 48, 49] or small molecules such as cyclodextrins [50]. Furthermore, the pore size of DNA‐scaffolded ALM nanopores could be altered by modifying the terminus of ALM attached to the DNA scaffold, producing pores large enough to interact with target molecules and thereby enabling single‐molecule sensing. Although the concept of tuning protein or peptide assembly with DNA has been proposed since 2017 [37, 38, 39], single‐molecule sensing with this class of pores has remained challenging. Our results represent the first demonstration of a functional DNA‐scaffolded peptide nanopore. The key strategy behind creating such a nanopore lies in controlling the packing of the peptide components. As we have shown here, looser packing appears advantageous, as the resulting pore inherently exhibits a larger diameter and can be further expanded by tuning the direction of the helix dipole–derived electrostatic force acting on the peptides.

We would also like to highlight two fundamental scientific findings from this study. First, we found that ALM favors hexameric aggregation. This possibility had previously been suggested by scanning tunneling microscopy imaging of ALM clusters on lipid membranes supported by gold substrates [55], as well as by MD simulations [66]. However, both of these approaches can yield results that differ from electrical recordings of single ALM channels. Another study templated ALM with α‐ or β‐cyclodextrins, and single‐channel formation was observed in both cases by electrical recording [50], but the authors did not further investigate ALM's oligomerization preference. In contrast, our results provide, for the first time, experimental evidence that ALM preferentially assembles into hexamers at the single‐channel level under an applied field. Second, we investigated how the helix dipole can influence peptide assembly, thereby producing channels with distinct geometries. Such dipoles have previously been reported to influence the aggregation of subunits in voltage‐gated K^+^ channels [68] and thus the findings of this study may help answer key questions in understanding ion channel mechanism.

Compared with previously established nanopores, our DNA‐scaffolded ALM pores also exhibited several limitations. (i) Achieving pores that simultaneously exhibit high insertion efficiency and sufficient size for molecular translocation is challenging. Because ALM preferentially inserts into lipid membranes from its N‐terminus, using this terminus to tune pore size by attaching it to DNA scaffolds makes the insertion of hexa‐NALM pores difficult. In our experiments, only ∼20% of trials resulted in stable pore insertion, meaning that a full day of experiments using multiple amplifiers yielded only a few usable datasets. (ii) The pore size of hexa‐NALM nanopores is not uniform. Since 3_10_‐helices are less stable than α‐helices, even small changes in the force balance acting on ALM can alter pore structure, as reflected by the large variation in conductance. Such variation is particularly unfavorable for nanopore sensing or sequencing applications. (iii) The effect of DNA scaffold design on pore quality remains poorly understood. This includes, first, whether the flexibility of the DNA scaffold affects peptide assembly. Previous simulations suggest that the arms of the DNA scaffold can adopt different orientations in the absence of external force [38]. Although lipid–peptide interactions are assumed to provide such forces and promote membrane insertion of all peptides, it is not yet known whether this occurs in real systems. Second, DNA scaffolds may hinder the entry of molecules into the pore. In a previous study, Fennouri et al. attributed the absence of translocation events in their DNA‐scaffolded CtxA nanopore to the presence of DNA scaffolds [39]. In contrast, in our case, we observed molecular translocation from both the DNA‐modified and unmodified sides of the hexa‐NALM nanopore. Further studies will be necessary to systematically investigate how DNA scaffold design influences both pore formation efficiency and molecular translocation through a combination of experimental approaches and MD simulations. In addition, although our nanopores are large enough to enable the translocation of unfolded DNA and peptides, further increasing their size to make them comparable to solid‐state nanopores and enable the translocation of folded molecules would be beneficial for future applications [79, 80, 81].

Overall, although the nanopores described in this study exhibit certain limitations, they mark an important step toward the initial realization of DNA‐hybridized peptide nanopores for functional nanopore sensing. We emphasize that these nanopores can be further advanced by exploiting the programmable nature of DNA, by harnessing the geometry of the 3_10_‐helix, and by introducing unnatural amino acids into peptides, thereby opening new horizons for the precise design of nanopores.

## Author Contributions

**Zugui Peng**: writing – original draft, writing – review and editing, project administration, funding acquisition, methodology, conceptualization, data curation, visualization, investigation, formal analysis. **Ryuji Kawano**: conceptualization, writing – review and editing, supervision, funding acquisition, resources. **Daisuke Noshiro**: investigation, writing – review and editing. **Shiroh Futaki**: supervision, writing – review and editing, resources.

## Conflicts of Interest

The authors declare no conflicts of interest.

## Supporting information



The data supporting the findings of this study are available in the Supporting Information of this article. The authors have cited additional references within the Supporting Information [46, 65, 71, 72, 82, 83, 84, 85, 86, 87]. **Supporting File**: anie72805‐sup‐0001‐SuppMat.pdf.

## Data Availability

The data that support the findings of this study are available in the Supporting Information of this article.

## References

[1] Y. L. Ying , Z. L. Hu , S. L. Zhang , et al., “Nanopore‐Based Technologies Beyond DNA Sequencing,” Nature Nanotechnology 17 (2022): 1136–1146, 10.1038/s41565-022-01193-2. 36163504

[2] A. Dorey and S. Howorka , “Nanopore DNA Sequencing Technologies and Their Applications Towards Single‐Molecule Proteomics,” Nature Chemistry 16 (2024): 314–334, 10.1038/s41557-023-01322-x. 38448507

[3] S. Takiguchi , N. Takeuchi , V. Shenshin , et al., “Harnessing DNA Computing and Nanopore Decoding for Practical Applications: From Informatics to microRNA‐Targeting Diagnostics,” Chemical Society Reviews 54 (2025): 8–32, 10.1039/D3CS00396E. 39471098 PMC11521203

[4] X. Lu , X. Du , D. Zhong , et al., “Nanopore Environmental Analysis,” JACS Au 5 (2025): 1570–1590, 10.1021/jacsau.5c00114. 40313842 PMC12042043

[5] K. Motone and J. Nivala , “Not if but When Nanopore Protein Sequencing Meets Single‐Cell Proteomics,” Nature Methods 20 (2023): 336–338, 10.1038/s41592-023-01800-7. 36899162

[6] J. Ritmejeris , X. Chen , and C. Dekker , “Single‐Molecule Protein Sequencing With Nanopores,” Nature Reviews Bioengineering 3 (2024): 303–316, 10.1038/s44222-024-00260-8.

[7] C. Lu , A. Bonini , J. H. Viel , and G. Maglia , “Toward Single‐Molecule Protein Sequencing Using Nanopores,” Nature Biotechnology 43 (2025): 312–322, 10.1038/s41587-025-02587-y. PMC12006967 40097683

[8] S. M. Bezrukov , I. Vodyanoy , and V. A. Parsegian , “Counting Polymers Moving Through a Single Ion Channel,” Nature 370 (1994): 279–281, 10.1038/370279a0. 7518571

[9] Y. Yi , P. Song , Z. Li , et al., “Nanopore‐Based Enzyme‐Linked Immunosorbent Assay for Cancer Biomarker Detection,” Nature Nanotechnology 20 (2025): 1079–1086, 10.1038/s41565-025-01918-z. 40369343

[10] Y. Qing and H. Bayley , “Enzymeless DNA Base Identification by Chemical Stepping in a Nanopore,” Journal of the American Chemical Society 143 (2021): 18181–18187, 10.1021/jacs.1c07497. 34669377

[11] W. Sun , Y. Xiao , K. Wang , et al., “Nanopore Discrimination of Rare Earth Elements,” Nature Nanotechnology 20 (2025): 523–531, 10.1038/s41565-025-01864-w. 39930101

[12] I. C. Nova , J. M. Craig , A. Mazumder , et al., “Nanopore Tweezers Show Fractional‐Nucleotide Translocation in Sequence‐Dependent Pausing by RNA Polymerase,” Proceedings of the National Academy of Sciences 121 (2024): e2321017121, 10.1073/pnas.2321017121. PMC11260103 38990947

[13] F. L. R. Lucas , K. Sarthak , E. M. Lenting , et al., “The Manipulation of the Internal Hydrophobicity of FraC Nanopores Augments Peptide Capture and Recognition,” ACS Nano 15 (2021): 9600–9613, 10.1021/acsnano.0c09958. 34060809 PMC8223486

[14] G. Huang , A. Voet , and G. Maglia , “FraC Nanopores With Adjustable Diameter Identify the Mass of Opposite‐Charge Peptides With 44 Dalton Resolution,” Nature Communications 10 (2019): 835, 10.1038/s41467-019-08761-6. PMC6381162 30783102

[15] M. Y. Li , H. Niu , J. Jiang , X. Y. Wu , Y. L. Ying , and Y. T. Long , “Real‐Time Recording the Dynamic Catalytic Heterogeneity of Enzymatic Reactions Using a Nanopore,” Journal of the American Chemical Society 147 (2025): 17121–17131, 10.1021/jacs.5c02535. 40354520

[16] M. Afshar Bakshloo , J. J. Kasianowicz , M. Pastoriza‐Gallego , et al., “Nanopore‐Based Protein Identification,” Journal of the American Chemical Society 144 (2022): 2716–2725, 10.1021/jacs.1c11758. 35120294

[17] K. Motone , D. Kontogiorgos‐Heintz , J. Wee , et al., “Multi‐Pass, Single‐Molecule Nanopore Reading of Long Protein Strands,” Nature 633 (2024): 662–669, 10.1038/s41586-024-07935-7. 39261738 PMC11410661

[18] S. Berhanu , S. Majumder , T. Muntener , et al., “Sculpting Conducting Nanopore Size and Shape Through De Novo Protein Design,” Science 385 (2024): 282–288, 10.1126/science.adn3796. 39024453 PMC11549965

[19] A. A. Vorobieva , P. White , B. Y. Liang , et al., “De Novo Design of Transmembrane β Barrels,” Science 371 (2021): eabc8182, 10.1126/science.abc8182. 33602829 PMC8064278

[20] Y. Li , B. S. Harris , Z. Li , et al., “Water, Solute, and Ion Transport in De Novo‐Designed Membrane Protein Channels,” ACS Nano 19 (2025): 2185–2195, 10.1021/acsnano.4c11317. 39714958 PMC13725935

[21] H. T. Kratochvil , L. C. Watkins , M. Mravic , et al., “Transient Water Wires Mediate Selective Proton Transport in Designed Channel Proteins,” Nature Chemistry 15 (2023): 1012–1021, 10.1038/s41557-023-01210-4. PMC10475958 37308712

[22] A. Niitsu , A. R. Thomson , A. J. Scott , et al., “Rational Design Principles for De Novo α‐Helical Peptide Barrels With Dynamic Conductive Channels,” Journal of the American Chemical Society 147 (2025): 11741–11753, 10.1021/jacs.4c13933. 40152328

[23] K. Shimizu , B. Mijiddorj , M. Usami , et al., “De Novo Design of a Nanopore for Single‐Molecule Detection That Incorporates a β‐Hairpin Peptide,” Nature Nanotechnology 17 (2022): 67–75, 10.1038/s41565-021-01008-w. PMC8770118 34811552

[24] R. S. Krishnan , K. Jana , A. H. Shaji , et al., “Assembly of Transmembrane Pores From Mirror‐Image Peptides,” Nature Communications 13 (2022): 5377. 10.1038/s41467-022-33155-6 PMC9474448 36104348

[25] K. R. Mahendran , A. Niitsu , L. B. Kong , et al., “A Monodisperse Transmembrane α‐Helical Peptide Barrel,” Nature Chemistry 9 (2017): 411–419, 10.1038/nchem.2647. 28430192

[26] Z. Peng , M. Usami , A. Nakada , et al., “De Novo Design of Alpha‐Helical Peptide Nanopores for Single‐Molecule Detection Using Helix Packing Motifs,” ACS Nano 19 (2025): 41789–41802, 10.1021/acsnano.5c15080. 41324174 PMC12713772

[27] Y. Xing , A. Dorey , L. Jayasinghe , and S. Howorka , “Highly Shape‐ and Size‐Tunable Membrane Nanopores Made With DNA,” Nature Nanotechnology 17 (2022): 708–713, 10.1038/s41565-022-01116-1. 35484212

[28] R. Zhang , Y. Xiang , and Y. Yang , “Passing Behavior of Oligonucleotides Through a Stacked DNA Nanochannel With Featured Path Design,” Journal of the American Chemical Society 146 (2024): 17122–17130, 10.1021/jacs.4c02734. 38861703

[29] H. Akai , T. Hirano , T. Mabuchi , and K. Shoji , “Specific ATP Detection Using Molecule‐Responsive DNA Nanopores,” Small 21 (2025): e2409293, 10.1002/smll.202409293. 40317998 PMC12288776

[30] S. Iwabuchi , I. Kawamata , S. Murata , and S. Nomura , “A Large, Square‐Shaped, DNA Origami Nanopore With Sealing Function on a Giant Vesicle Membrane,” Chemical Communications 57 (2021): 2990–2993, 10.1039/D0CC07412H. 33587063

[31] J. Burns , A. Seifert , N. Fertig , and S. Howorka , “A Biomimetic DNA‐Based Channel for the Ligand‐Controlled Transport of Charged Molecular Cargo Across a Biological Membrane,” Nature Nanotechnology 11 (2016): 152–156, 10.1038/nnano.2015.279. 26751170

[32] S. Krishnan R , N. Puthumadathil , A. H. Shaji , K. Santhosh Kumar , G. Mohan , and K. R. Mahendran , “Designed Alpha‐Helical Barrels for Charge‐Selective Peptide Translocation,” Chemical Science 12 (2020): 639–649. 34163795 10.1039/d0sc04856a PMC8178987

[33] R. Satheesan , D. Vikraman , P. Jayan , V. Vijayan , C. Chimerel , and K. R. Mahendran , “Sensing PEGylated Peptide Conformations Using a Protein Nanopore,” Nano Letters 24 (2024): 3566–3574, 10.1021/acs.nanolett.3c03247. 38316144

[34] N. Firzan Ca , K. Jana , S. Radhakrishnan , et al., “Fabrication of Cytotoxic Mirror Image Nanopores,” Nature Communications 16 (2025): 8666, 10.1038/s41467-025-64025-6. PMC12491518 41038852

[35] S. Fujita , I. Kawamura , and R. Kawano , “Cell‐Free Expression of De Novo Designed Peptides That Form β‐Barrel Nanopores,” ACS Nano 17 (2023): 3358–3367, 10.1021/acsnano.2c07970. 36731872 PMC9979648

[36] M. Serra‐Batiste , M. Ninot‐Pedrosa , M. Bayoumi , M. Gairi , G. Maglia , and N. Carulla , “Aβ42 Assembles Into Specific β‐Barrel Pore‐Forming Oligomers in Membrane‐Mimicking Environments,” Proceedings of the National Academy of Sciences of the United States of America 113 (2016): 10866–10871, 10.1073/pnas.1605104113. 27621459 PMC5047179

[37] A. Henning‐Knechtel , J. Knechtel , and M. Magzoub , “DNA‐Assisted Oligomerization of Pore‐Forming Toxin Monomers into Precisely‐Controlled Protein Channels,” Nucleic Acids Research 45 (2017): 12057–12068, 10.1093/nar/gkx990. 29088457 PMC5716084

[38] E. Spruijt , S. E. Tusk , and H. Bayley , “DNA Scaffolds Support Stable and Uniform Peptide Nanopores,” Nature Nanotechnology 13 (2018): 739–745, 10.1038/s41565-018-0139-6. 29808001

[39] A. Fennouri , J. List , J. Ducrey , et al., “Tuning the Diameter, Stability, and Membrane Affinity of Peptide Pores by DNA‐Programmed Self‐Assembly,” ACS Nano 15 (2021): 11263–11275, 10.1021/acsnano.0c10311. 34128638

[40] Q. Shen , Q. Xiong , K. Zhou , et al., “Functionalized DNA‐Origami‐Protein Nanopores Generate Large Transmembrane Channels With Programmable Size‐Selectivity,” Journal of the American Chemical Society 145 (2023): 1292–1300, 10.1021/jacs.2c11226. 36577119 PMC9852090

[41] M. Soskine , A. Biesemans , B. Moeyaert , S. Cheley , H. Bayley , and G. Maglia , “An Engineered ClyA Nanopore Detects Folded Target Proteins by Selective External Association and Pore Entry,” Nano Letters 12 (2012): 4895–4900, 10.1021/nl3024438. 22849517 PMC3440510

[42] J. Walshaw and D. N. Woolfson , “Extended Knobs‐Into‐Holes Packing in Classical and Complex Coiled‐Coil Assemblies,” Journal of Structural Biology 144 (2003): 349–361, 10.1016/j.jsb.2003.10.014. 14643203

[43] R. F. S. Walters and W. F. DeGrado , “Helix‐Packing Motifs in Membrane Proteins,” Proceedings of the National Academy of Sciences of the United States of America 103 (2006): 13658–13663, 10.1073/pnas.0605878103. 16954199 PMC1564267

[44] T. Nagao , D. Mishima , N. Javkhlantugs , et al., “Structure and Orientation of Antibiotic Peptide Alamethicin in Phospholipid Bilayers as Revealed by Chemical Shift Oscillation Analysis of Solid State Nuclear Magnetic Resonance and Molecular Dynamics Simulation,” Biochimica Et Biophysica Acta 1848 (2015): 2789–2798, 10.1016/j.bbamem.2015.07.019. 26248014

[45] H. Duclohier and H. Wroblewski , “Voltage‐Dependent Pore Formation and Antimicrobial Activity by Alamethicin and Analogues,” Journal of Membrane Biology 184 (2001): 1–12, 10.1007/s00232-001-0077-2. 11687873

[46] T. Okazaki , M. Sakoh , Y. Nagaoka , and K. Asami , “Ion Channels of Alamethicin Dimer N‐Terminally Linked by Disulfide Bond,” Biophysical Journal 85 (2003): 267–273, 10.1016/S0006-3495(03)74472-5. 12829482 PMC1303083

[47] D. Noshiro , K. Asami , and S. Futaki , “Metal‐Assisted Channel Stabilization: Disposition of a Single Histidine on the N‐Terminus of Alamethicin Yields Channels With Extraordinarily Long Lifetimes,” Biophysical Journal 98 (2010): 1801–1808, 10.1016/j.bpj.2010.01.028. 20441743 PMC2862188

[48] S. Futaki , M. Fukuda , M. Omote , et al., “Alamethicin−Leucine Zipper Hybrid Peptide: A Prototype for the Design of Artificial Receptors and Ion Channels,” Journal of the American Chemical Society 123 (2001): 12127–12134, 10.1021/ja011166i. 11734010

[49] T. Kiwada , K. Sonomura , Y. Sugiura , K. Asami , and S. Futaki , “Transmission of Extramembrane Conformational Change Into Current: Construction of Metal‐Gated Ion Channel,” Journal of the American Chemical Society 128 (2006): 6010–6011, 10.1021/ja060515b. 16669650

[50] C. U. Hjorringgaard , B. S. Vad , V. V. Matchkov , et al., “Cyclodextrin‐Scaffolded Alamethicin With Remarkably Efficient Membrane Permeabilizing Properties and Membrane Current Conductance,” Journal of Physical Chemistry B 116 (2012): 7652–7659, 10.1021/jp2098679. 22676384

[51] L. Mereuta , A. Asandei , I. Schiopu , J. Park , Y. Park , and T. Luchian , “Synthetic Receptor Based on a Peptide Antibiotic‐Functionalized Chimera for Hybridization‐Based Polynucleotide Detection,” ACS Applied Materials & Interfaces 15 (2023): 33159–33168, 10.1021/acsami.3c06086. 37383014

[52] B. Leitgeb , A. Szekeres , L. Manczinger , C. Vagvolgyi , and L. Kredics , “The History of Alamethicin: A Review of the Most Extensively Studied Peptaibol,” Chemistry and Biodiversity 4 (2007): 1027–1051, 10.1002/cbdv.200790095. 17589875

[53] J. Breed , I. D. Kerr , R. Sankararamakrishnan , and M. S. Sansom , “Packing Interactions of Aib‐Containing Helices: Molecular Modeling of Parallel Dimers of Simple Hydrophobic Helices and of Alamethicin,” Biopolymers 35 (1995): 639–655, 10.1002/bip.360350610. 7766829

[54] R. Appavu and D. Mohan , “Fundamental of Secondary Structures in Peptide Based Synthetic Nanovaccine Development,” Transcriptomics: Open Access 4 (2016): 131, 10.4172/2329-8936.1000131.

[55] P. Pieta , J. Mirza , and J. Lipkowski , “Direct Visualization of the Alamethicin Pore Formed in a Planar Phospholipid Matrix,” Proceedings of the National Academy of Sciences 109 (2012): 21223–21227, 10.1073/pnas.1201559110. PMC3535624 23236158

[56] M. S. Sansom , “The Biophysics of Peptide Models of Ion Channels,” Progress in Biophysics and Molecular Biology 55 (1991): 139–235, 10.1016/0079-6107(91)90004-C. 1715999

[57] R. Kawano , Y. Tsuji , K. Kamiya , et al., “A Portable Lipid Bilayer System for Environmental Sensing With a Transmembrane Protein,” PLoS One 9 (2014): e102427, 10.1371/journal.pone.0102427. 25072468 PMC4114450

[58] H. Watanabe , A. Gubbiotti , M. Chinappi , et al., “Analysis of Pore Formation and Protein Translocation Using Large Biological Nanopores,” Analytical Chemistry 89 (2017): 11269–11277, 10.1021/acs.analchem.7b01550. 28980803

[59] M. Akeson , D. Branton , J. J. Kasianowicz , E. Brandin , and D. W. Deamer , “Microsecond Time‐Scale Discrimination Among Polycytidylic Acid, Polyadenylic Acid, and Polyuridylic Acid as Homopolymers or as Segments Within Single RNA Molecules,” Biophysical Journal 77 (1999): 3227–3233, 10.1016/S0006-3495(99)77153-5. 10585944 PMC1300593

[60] Y. L. Ying , D. W. Li , Y. Li , J. S. Lee , and Y. T. Long , “Enhanced Translocation of Poly(dt)45 Through an α‐hemolysin Nanopore by Binding With Antibody,” Chemical Communications (Cambridge) 47 (2011): 5690, 10.1039/c0cc05787h. 21491051

[61] Y. Wang , K. Tian , L. L. Hunter , B. Ritzo , and L. Q. Gu , “Probing Molecular Pathways for DNA Orientational Trapping, Unzipping and Translocation in Nanopores by Using a Tunable Overhang Sensor,” Nanoscale 6 (2014): 11372–11379, 10.1039/C4NR03195D. 25144935 PMC6201287

[62] M. Miyagi , S. Takiguchi , K. Hakamada , M. Yohda , and R. Kawano , “Single Polypeptide Detection Using a Translocon EXP2 Nanopore,” Proteomics 22 (2022): e2100070, 10.1002/pmic.202100070. 34411416

[63] H. Ouldali , K. Sarthak , T. Ensslen , et al., “Electrical Recognition of the Twenty Proteinogenic Amino Acids Using an Aerolysin Nanopore,” Nature Biotechnology 38 (2020): 176–181, 10.1038/s41587-019-0345-2. PMC7008938 31844293

[64] G. Maglia , M. R. Restrepo , E. Mikhailova , and H. Bayley , “Enhanced Translocation of Single DNA Molecules Through α‐Hemolysin Nanopores by Manipulation of Internal Charge,” Proceedings of the National Academy of Sciences 105 (2008): 19720–19725, 10.1073/pnas.0808296105. PMC2604925 19060213

[65] G. Di Muccio , A. E. Rossini , D. Di Marino , G. Zollo , and M. Chinappi , “Insights Into Protein Sequencing With an α‐Hemolysin Nanopore by Atomistic Simulations,” Scientific Reports 9 (2019): 6440, 10.1038/s41598-019-42867-7. 31015503 PMC6478933

[66] C. Wei and A. Pohorille , “Multi‐Oligomeric States of Alamethicin Ion Channel: Assemblies and Conductance,” Biophysical Journal 122 (2023): 2531–2543, 10.1016/j.bpj.2023.05.006. 37161094 PMC10323028

[67] S. F. Mayer , M. F. Mitsioni , P. Robin , et al., “Lumen Charge Governs Gated Ion Transport in Beta‐Barrel Nanopores,” Nature Nanotechnology 21 (2025): 116–124. 10.1038/s41565-025-02052-6 PMC12819157 41219410

[68] L. Adhya , T. Mapder , and S. Adhya , “Role of Terminal Dipole Charges in Aggregation of α‐Helix Pair in the Voltage Gated K+ Channel,” Biochimica Et Biophysica Acta 1828 (2013): 845–850, 10.1016/j.bbamem.2012.11.008. 23159811

[69] F. L. R. Lucas , R. C. A. Versloot , L. Yakovlieva , M. T. C. Walvoort , and G. Maglia , “Protein Identification by Nanopore Peptide Profiling,” Nature Communications 12 (2021): 5795, 10.1038/s41467-021-26046-9. PMC8490355 34608150

[70] C. Cao , D. F. Liao , J. Yu , H. Tian , and Y. T. Long , “Construction of an Aerolysin Nanopore in a Lipid Bilayer for Single‐Oligonucleotide Analysis,” Nature Protocols 12 (2017): 1901–1911, 10.1038/nprot.2017.077. 28837133

[71] M. Z. Huo , M. Y. Li , Y. L. Ying , and Y. T. Long , “Is the Volume Exclusion Model Practicable for Nanopore Protein Sequencing?,” Analytical Chemistry 93 (2021): 11364–11369, 10.1021/acs.analchem.1c00851. 34379401

[72] M. Y. Li , Y. L. Ying , J. Yu , et al., “Revisiting the Origin of Nanopore Current Blockage for Volume Difference Sensing at the Atomic Level,” JACS Au 1 (2021): 967–976, 10.1021/jacsau.1c00109. 34467343 PMC8395674

[73] M. Chinappi , M. Yamaji , R. Kawano , and F. Cecconi , “Analytical Model for Particle Capture in Nanopores Elucidates Competition Among Electrophoresis, Electroosmosis, and Dielectrophoresis,” ACS Nano 14 (2020): 15816–15828, 10.1021/acsnano.0c06981. 33170650 PMC8016366

[74] M. Baldelli , G. Di Muccio , A. Sauciuc , et al., “Controlling Electroosmosis in Nanopores Without Altering the Nanopore Sensing Region,” Advanced Materials 36 (2024): e2401761, 10.1002/adma.202401761. 38860821

[75] S. L. Zhang , G. Huang , R. C. A. Versloot , et al., “Bottom‐up Fabrication of a Proteasome–Nanopore That Unravels and Processes Single Proteins,” Nature Chemistry 13 (2021): 1192–1199, 10.1038/s41557-021-00824-w. PMC7612055 34795436

[76] S. Straathof , G. Di Muccio , and G. Maglia , “Nanopores With an Engineered Selective Entropic Gate Detect Proteins at Nanomolar Concentration in Complex Biological Sample,” Journal of the American Chemical Society 147 (2025): 15050–15065, 10.1021/jacs.4c17147. 40261977 PMC12063177

[77] F. Gao , J. H. Wang , H. Ma , et al., “Identification of Oligosaccharide Isomers Using Electrostatically Asymmetric OmpF Nanopore,” Angewandte Chemie International Edition 64 (2025): e202422118, 10.1002/anie.202422118. 39856493

[78] M. Ryu , S. Oh , K. B. Jeong , et al., “Single‐Molecule‐Based, Label‐Free Monitoring of Molecular Glue Efficacies for Promoting Protein–Protein Interactions Using YaxAB Nanopores,” ACS Nano 18 (2024): 31451–31465, 10.1021/acsnano.4c11436. 39482865 PMC11562796

[79] Z. Q. Zhou , S. C. Liu , J. Wang , et al., “Exploring a Solid‐State Nanopore Approach for Single‐Molecule Protein Detection From Single Cells,” Chemical Science 16 (2025): 8501–8508, 10.1039/D5SC01764E. 40236591 PMC11996040

[80] S. C. Liu , Y. L. Ying , W. H. Li , Y. J. Wan , and Y. T. Long , “Snapshotting the Transient Conformations and Tracing the Multiple Pathways of Single Peptide Folding Using a Solid‐State Nanopore,” Chemical Science 12 (2021): 3282–3289, 10.1039/D0SC06106A. 34164097 PMC8179386

[81] K. L. Chen , Y. L. Ying , A. G. Ewing , and Y. T. Long , “Nanopipette Electrochemistry,” Chemical Reviews 126 (2026): 149–183, 10.1021/acs.chemrev.5c00454. 41416710

[82] S. Iwabuchi , S. Nomura , and Y. Sato , “Surfactant‐Assisted Purification of Hydrophobic DNA Nanostructures,” ChemBioChem 24 (2023): e202200568, 10.1002/cbic.202200568. 36470849

[83] Y. Sekiya , K. Shimizu , Y. Kitahashi , A. Ohyama , I. Kawamura , and R. Kawano , “Electrophysiological Analysis of Membrane Disruption by Bombinin and its Isomer Using the Lipid Bilayer System,” ACS Applied Bio Materials 2 (2019), 1542–1548, 10.1021/acsabm.8b00835. 35026927

[84] M. Ohara , M. Takinoue , and R. Kawano , “Nanopore Logic Operation With DNA to RNA Transcription in a Droplet System,” ACS Synthetic Biology 6 (2017): 1427–1432, 10.1021/acssynbio.7b00101. 28414903

[85] B. Hille , Ion Channels of Excitable Membranes, 3rd ed. (Sinauer, 2001).

[86] M. Pastoriza‐Gallego , M. F. Breton , F. Discala , L. Auvray , J. M. Betton , and J. Pelta , “Evidence of Unfolded Protein Translocation Through a Protein Nanopore,” ACS Nano 8 (2014): 11350–11360, 10.1021/nn5042398. 25380310

[87] C. Cao , Y. L. Ying , Z. L. Hu , D. F. Liao , H. Tian , and Y. T. Long , “Discrimination of Oligonucleotides of Different Lengths With a Wild‐Type Aerolysin Nanopore,” Nature Nanotechnology 11 (2016): 713–718, 10.1038/nnano.2016.66. 27111839

