# Supplementary material for: Large‐Diameter DNA‐Scaffolded Nanopores Enabled by Loosely Packed Peptides for Single‐Molecule Sensing
Source: Angew Chem Int Ed Engl. 2026 May 22;65(29):e3311099. doi: 10.1002/anie.3311099 (PMC13360634; doi:10.1002/anie.3311099)
Supplement: Supplementary file 1 — The data supporting the findings of this study are available in the Supporting Information of this article. The authors have cited additional references within the Supporting Information [46, 65, 71, 72, 82, 83, 84, 85, 86, 87]. Supporting File: anie72805‐sup‐0001‐SuppMat.pdf. [file ANIE-65-e3311099-s001.pdf]

Supporting Information

Large-Diameter DNA-Scaffolded Nanopores Enabled by Loosely Packed Peptides for Single-Molecule Sensing

Zugui Peng, [a] [d] Daisuke Noshiro,[b] Shiroh Futaki,[c] and Ryuji Kawano*[a]

[a] Dr. Z. Peng, Prof. R. Kawano
Department of Biotechnology and Life Science
Tokyo University of Agriculture and Technology
2-24-16 Naka-cho, Koganei-shi, Tokyo 184-8588, Japan
E-mail: rjkawano@cc.tuat.ac.jp

[b] Dr. D. Noshiro
Institute for Genetic Medicine
Hokkaido University
Sapporo, Hokkaido 060-0815, Japan

[c] Prof. S. Futaki
Graduate School of Pharmaceutical Sciences
Kyoto University
Sakyo-ku, Kyoto 606-8501, Japan

[d] Dr. Z. Peng
School of Engineering
Institute of Science Tokyo
Meguro-ku, Tokyo 152-8550, Japan

Experimental Section

General: The following reagents were used: 1,2-diphytanoyl-sn-glycero-3-phosphocholine (DPhPC, Avanti Polar Lipids, USA); dibenzocyclooctyne-sulfo-N-hydroxysuccinimidyl ester (Sigma-Aldrich, USA); 40(w/v)% acrylamide/bis mixed solution (29:1, Nacalai Tesque, Japan); SYBR™ Green II Nucleic Acid Gel Stain (Takara Bio, Japan); poly-L-lysine hydrobromide (PLL, Mw=30,000-70,000, Sigma-Aldrich, USA); sodium dodecyl sulfate (SDS, FUJIFILM Wako Pure Chemical Industries, Japan); n-dodecyl β-d-maltoside (DDM, FUJIFILM Wako Pure Chemical Industries, Japan); N,N,N',N'-tetramethylethylenediamine (TEMED, FUJIFILM Wako Pure Chemical Industries, Japan), ammonium persulfate (APS, FUJIFILM Wako Pure Chemical Industries, Japan), decane (FUJIFILM Wako Pure Chemical Industries, Japan); dimethyl sulfoxide (DMSO, FUJIFILM Wako Pure Chemical Industries, Japan); methanol (Kishida Chemical, Japan); triethylammonium acetate (TEAA, Tokyo Chemical Industry, Japan); ethylenediaminetetraacetic acid (EDTA, NIPPON GENE, Japan); tris(hydroxymethyl)aminomethane (Tris, FUJIFILM Wako Pure Chemical Industries, Japan); 3-(N-morpholino)propanesulfonic acid (MOPS, Nacalai Tesque, Japan); boric acid (FUJIFILM Wako Pure Chemical Industries, Japan); potassium dihydrogen phosphate (KH_2PO_4, FUJIFILM Wako Pure Chemical Industries, Japan); magnesium chloride ($MgCl_2$, Nacalai Tesque, Japan); potassium chloride (KCl, Nacalai Tesque, Japan).

Peptides: WT-ALM from *Trichoderma viride* was purchased from Sigma-Aldrich (USA). CAZ-ALM and NAZ-ALM were obtained through custom peptide synthesis by Cosmo Bio (Japan). The two peptides were purified by HPLC to >90% purity. R7G peptide was a kind gift from the laboratory of Izuru Kawamura at Yokohama National University. di-ALM was synthesized manually by solid-phase following a previous report.[46] Briefly, o-chlorotrityl resin was used as the solid support. To assemble the sterically hindered Aib residues and amino acids adjacent to Aib, the corresponding Fmoc–amino acid fluorides, which were prepared from the Fmoc–amino acids using cyanuryl fluoride or (diethylamino)sulfur trifluoride were employed. All other amino acids were coupled using a Fmoc-based solid-phase peptide synthesis protocols. After completion of amino acid assembly, the resin

was further reacted with S-acetamidomethyl-β-mercaptopropionic acid and subsequently cleaved to release the peptide. The crude peptide was purified by gel chromatography followed by RP-HPLC. The purified product was then treated with 0.025 M I_2 in 70–80% CH_3CN/H_2O to remove the acetamidomethyl group and induce disulfide bond formation, yielding di-ALM, which was finally purified by gel chromatography or RP-HPLC.

DNA Modification: 5′-Amino Modifier C6–modified DNA oligonucleotides, purified by standard desalting, were purchased from Integrated DNA Technologies (USA). To attach a DBCO group to the DNA terminus, 250 μM DNA was reacted with 5 mM dibenzocyclooctyne-sulfo-N-hydroxysuccinimidyl ester in 100 mM KH_2PO_4 containing 30% (v/v) DMSO (pH 8.0) for 6 h at 37 °C. The DBCO-modified DNA was then purified by RP-HPLC using a JASCO EXTREMA system equipped with a Unifinepak C18 column (5 μm, 4.6 mm × 250 mm). Elution was performed with an isocratic hold of buffer A for 5 min, followed by a linear gradient to 40% buffer B over 20 min at a flow rate of 1.0 mL/min at 40 °C (buffer A: 50 mM TEAA, pH 7.0; buffer B: methanol). After RP-HPLC purification, the solvent of the resulting solution was removed using a EYELA CVE-3000 centrifugal evaporator, and the DNA was redissolved in Milli-Q water to a final concentration of 30 μM.

DNA-ALM conjugation: DNA scaffolds were preassembled prior to conjugation with ALM peptides. Equimolar amounts of each DBCO-modified oligonucleotide were mixed to a final concentration of 2 μM in TE20 buffer (10 mM Tris, 1 mM EDTA, 20 mM $MgCl_2$, pH 8.0). The mixture was heated at 95 °C for 5 min, immediately cooled to 65 °C, and then gradually cooled to 25 °C over 200 min. DNA–peptide conjugation was performed by mixing the preassembled DNA scaffolds with ALM peptides to achieve a final DNA scaffold concentration of 1.5 μM and a peptide concentration of 100 μM in a solution containing 50 mM DDM, followed by incubation at 37 °C overnight. The conjugation efficiency between single DNA strands and peptides was confirmed by RP-HPLC using a Unifinepak C18 column with a linear gradient from 100% buffer A to 100% buffer B over 40 min at a flow rate of 1.0 mL/min and a column temperature of 40 °C (buffer A: 50 mM TEAA, pH 7.0; buffer B: methanol).

DNA-ALM nanopore purification: A modified protocol[82] of polyacrylamide gel electrophoresis (PAGE) was used to purify the DNA-scaffolded ALM nanopores. Briefly, 5 μL of the reaction solution was loaded onto a homemade 5% polyacrylamide gel prepared with a running buffer containing 10 mM $MgCl_2$, 0.1% (w/v) SDS, and 1× TBE (89 mM Tris, 89 mM boric acid, 2 mM EDTA). Electrophoresis was performed at 120 V for 80 min. After electrophoresis, the gel was first washed with TBE containing 10 mM $MgCl_2$, then stained with SYBR™ Green II for 10 min, and imaged using an Atto LuminoGraph I imager. The gel band corresponding to the DNA–ALM nanopores was excised and soaked in 10 μL of TE20 buffer supplemented with 2 mM DDM, followed by incubation at 37 °C overnight. The solution was recovered by filtration using Ultrafree-MC centrifugal filters (Merck, Germany). The nanopore extract solution was stored at 25 °C before use.

Conjugation-first, assembly-later DNA–ALM nanopore formation: DBCO-modified DNAs (100 μM) were first reacted with ALM peptides (150 μM) individually in 100 mM K_2HPO_4 containing 30% (v/v) DMSO (pH 7.0) for 6 h at 37 °C. The peptide–DNA conjugates were purified by 10% denaturing (8 M urea) PAGE at 120 V for 100 min, excised from the gel, soaked in water, and further concentrated and purified using an Amicon Ultra-0.5 centrifugal filter unit (10 kDa, Millipore). Equimolar amounts of each ALM-modified oligonucleotide were mixed to a final concentration of 1 μM in TE20 buffer and then annealed using the same protocol as in the assembly-first, conjugation-later approach. The molecular weights were verified by 5% SDS–PAGE and the gel was stained with SYBR™ Green II.

Electrical recording and data analysis: Bilayer lipid membranes (BLMs) were prepared by the droplet contact method using microdevices at 22 ± 2 °C.[83,84] Bilayers were prepared by first treating an aperture (~100 μm in diameter) between *cis* and *trans* compartments with DPhPC solution (lipids/decane, 20 mg/mL, 0.2 μL). The *trans* compartment was then filled with buffer solution (1 M KCl, 10 mM MOPS, 5 μL, pH 7.0), and the *cis* compartment was filled with buffer solution (1 M KCl, 10 mM MOPS, 4.8 μL, pH 7.0), followed by the addition of 0.2 μL of nanopore extract solution. Channel currents were amplified using a Pico patch clamp amplifier (Tecella, USA) with an 8 kHz low-pass

filter at a sampling frequency of 40 kHz. For single-molecule sensing experiments, target molecules were premixed with the *trans* or *cis* solution to reach the desired final concentration before lipid bilayer formation. Data analysis was performed using pCLAMP ver. 11.0.3 (Molecular Devices, USA) and Excel (Microsoft, USA) software.

The diameter of the nanopores formed was calculated by the conductance of the step-like and square-top signals using Hille's equation,[85]

$$\frac{1}{G} = \left(L + \frac{\pi d}{4}\right)\frac{4\rho}{\pi d^2} \qquad (1)$$

where G, L, d, and ρ in the equation represent conductance, pore length, pore diameter, and solvent resistivity, respectively. The length of the pore was calculated assuming a helix length of 4 nm.

The RMS noise is calculated based on the following:

$$RMS = \sqrt{\frac{\sum_{i=1}^{n}(x_i - x_n)^2}{n}} \qquad (2)$$

Where *n* is the number of data points recorded, x_i is the respective current value, and x_m is the average current value. The RMS noise was calculated only from the first-step current trace observed during each recording, and therefore corresponds to single-pore events.

Supplementary tables

Table S1. Sequences of peptides used for nanopore formation. U, α-aminoisobutyric acid; X, N^2-(((9H-fluoren-9-yl)methoxy)carbonyl)-N^6-diazo-L-lysine; Pheol, phenylalaninol; Mp, 3-mercaptopropionic acid; Ac, acetyl.

Peptide	Sequences
**WT-ALM**	Ac-UPUAUAQUVGLUPVUUEQ-Pheol
**di-ALM**	Mp-UPUAUAQUVGLUPVUUEQ-Pheol
**CAZ-ALM**	Ac-UPUAUAQUVGLUPVUUEQFX-OH
**NAZ-ALM**	N_3-UPUAUAQUVGLUPVUUEQF-OH

Table S2. Estimated pore diameters calculated using the Hille equation.

Nanopore	Conductance [nS]	Diameter [nm]
**6-mer WT-ALM**	1.33	0.84
**7-mer WT-ALM**	2.63	1.22
**8-mer WT-ALM**	3.99	1.54
**6-mer di-ALM**	1.25	0.81
**8-mer di-ALM**	4.09	1.56
**hexa-CALM**	1.12	0.77
**dodeca-CALM**	5.8	1.91
**hexa-NALM**	2.5	1.18

Table S3. ID, sequences and chemical modification of the DNA strands used for constructing the DNA scaffolds.

ID	Sequences (5' → 3')
1	/5AmMC6/TTTGCCTCGAATCTTCTCCACTGAATCCATCCTCTTGTTTCTTGTGAAC
2	/5AmMC6/TTTTGCCATAAGTTTTTCAGTGGAGTCAGCAACATAGTTCTCTCAACAA
3	/5AmMC6/TTTGTTCACAAGATTTCGAAACCAATTGTTAGTGTAGTTGTGCATAAGC
4	/5AmMC6/TTTCCAACTGGGATTTTGGTTTCGATCAAGAGGATGGTTACTTATGGCA
5	/5AmMC6/TTTGCTTATGCACTTGAGTCACAGATCTATGTTGCTGTTGATTCGAGGC
6	/5AmMC6/TTTTTGTTGAGAGTTTCTGTGACTCTCTACACTAACATTTCCCAGTTGG
7	/5AmMC6/TTTGCTTATGCACTTGAGTCACAGATACGGGAAGCAGTTCTATGTTGCTGTTGATTCGAGGC
8	/5AmMC6/TTTTTGTTGAGAGTTCTGCTTCCCGTTTTATTGCTCGA
9	/5AmMC6/TTTGCTTATGCACTTGAGTCACAGATACGGGAAGCAGTTAACGTGTGAG
10	/5AmMC6/TTTTCGAGCAATATTTCTGTGACTCTCTACACTAACATTTCCCAGTTGG
11	/5AmMC6/TTTCTCACACGTTTTGATACGGACATCTATGTTGCTGTTGATTCGAGGC
12	/5AmMC6/TTTTTGTTGAGAGTTTGTCCGTATCTCTGCTTCCCGTTTTATTGCTCGA
13	/5AmMC6/TTTCTCACACGTTTTGATACGGACATCTTGGATAGCGTTAAAGCACCTC
14	/5AmMC6/TTTCACTTCACTTTTTGTCCGTATCTCTGCTTCCCGTTTTATTGCTCGA

**15**	/5AmMC6/TTTGAGGTGCTTTTTTGTCAATCGGTAGTAGCCTAGCTTAGCCTTAGC C
**16**	/5AmMC6/TTTGAAACAGATATTCCGATTGACACTGCTATCCAAGTTAAGTGAAGTG
**17**	/5AmMC6/TTTGGCTAAGGCTTTAATGAGTACCTCTATGTTGCTGTTGATTCGAGG C
**18**	/5AmMC6/TTTTTGTTGAGAGTTGGTACTCATTTGCTAGGCTACTTTTATCTGTTTC

Table S4. Names and composition of DNA scaffolds

Scaffold	ssDNA used
**hexascaffold**	1, 2, 3, 4, 5, 6
**heptascaffold**	1, 2, 3, 4, 7, 8, 10
**octascaffold**	1, 2, 3, 4, 9, 10, 11, 12
**dodascaffold**	1, 2, 3, 4, 9, 10, 13, 14, 15, 16, 17, 18

Supplementary figures

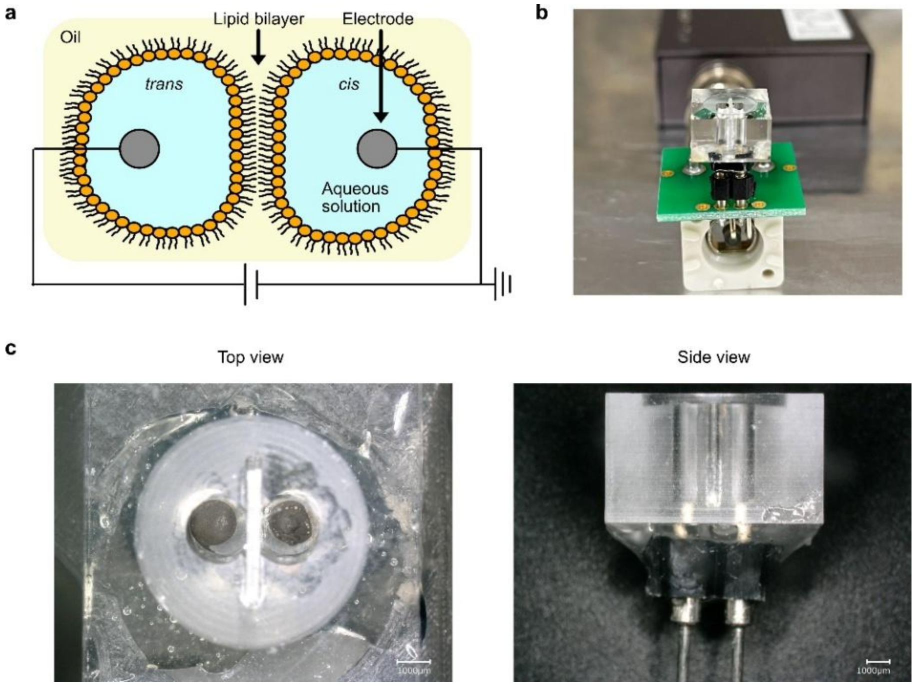

Figure S1. (a) Schematic illustration of the droplet contact method. (b) Overall view of the microdevice used for electrical recording experiments. (c) Enlarged top and side views of the microdevice.

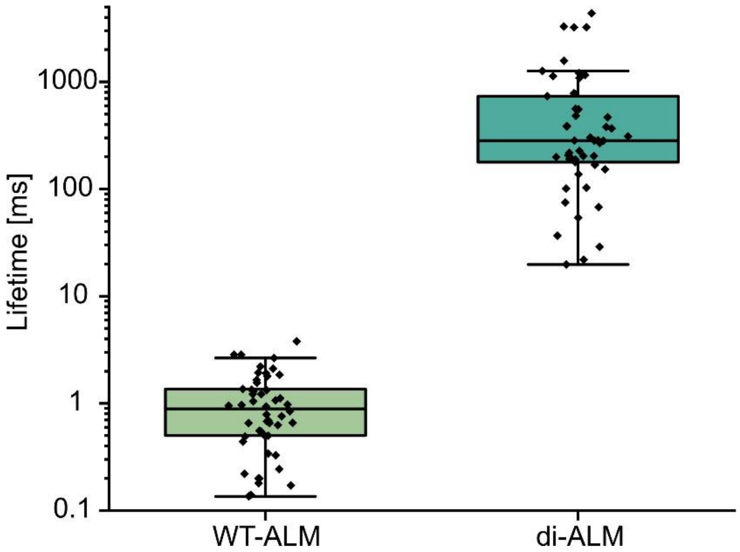

Figure S2. Lifetime of WT-ALM and di-ALM nanopores. Each dot represents an individual pore (n = 50). Error bars indicate the SD.

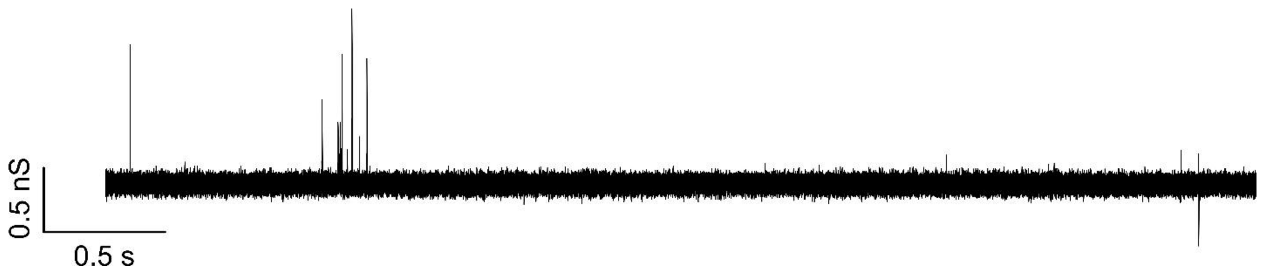

Figure S3. Electrical recordings of 1 μM WT-ALM at +100 mV. No distinguishable pore-formation signals were observed.

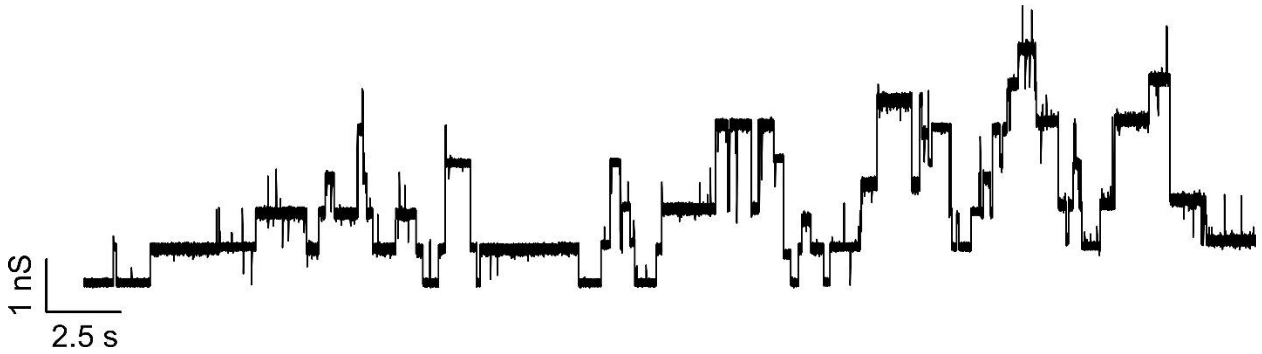

Figure S4. Prolonged electrical recordings of 100 nM di-ALM under an applied voltage of +100 mV. The lifetimes of the nanopores were only a few seconds, and additional pores subsequently opened.

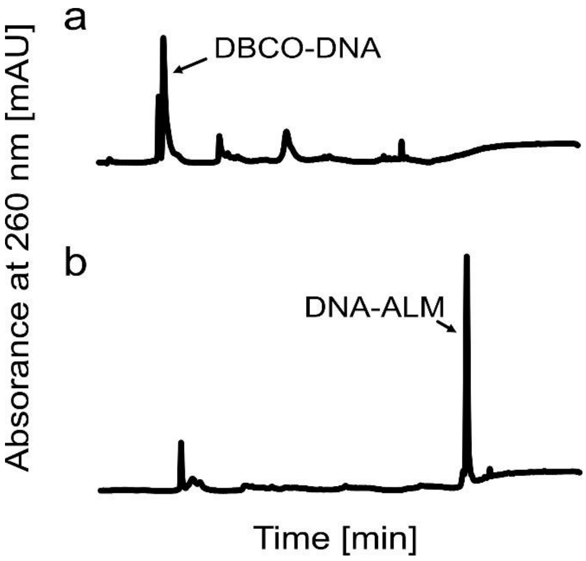

Figure S5. Analytical RP-HPLC chromatograms of (a) DBCO-modified DNA and (b) DNA reacted with CAZ-ALM for 6 h. For the reaction, 10 μM ssDNA **1** was incubated with 50 μM CAZ-ALM in TE20 buffer overnight. HPLC conditions: flow rate, 1.0 mL/min; temperature, 25 °C; mobile phase A, 100 mM triethylammonium acetate (TEAA), pH 7.0; mobile phase B, methanol. A linear gradient of phase B at 2.5%/min was applied for 40 min.

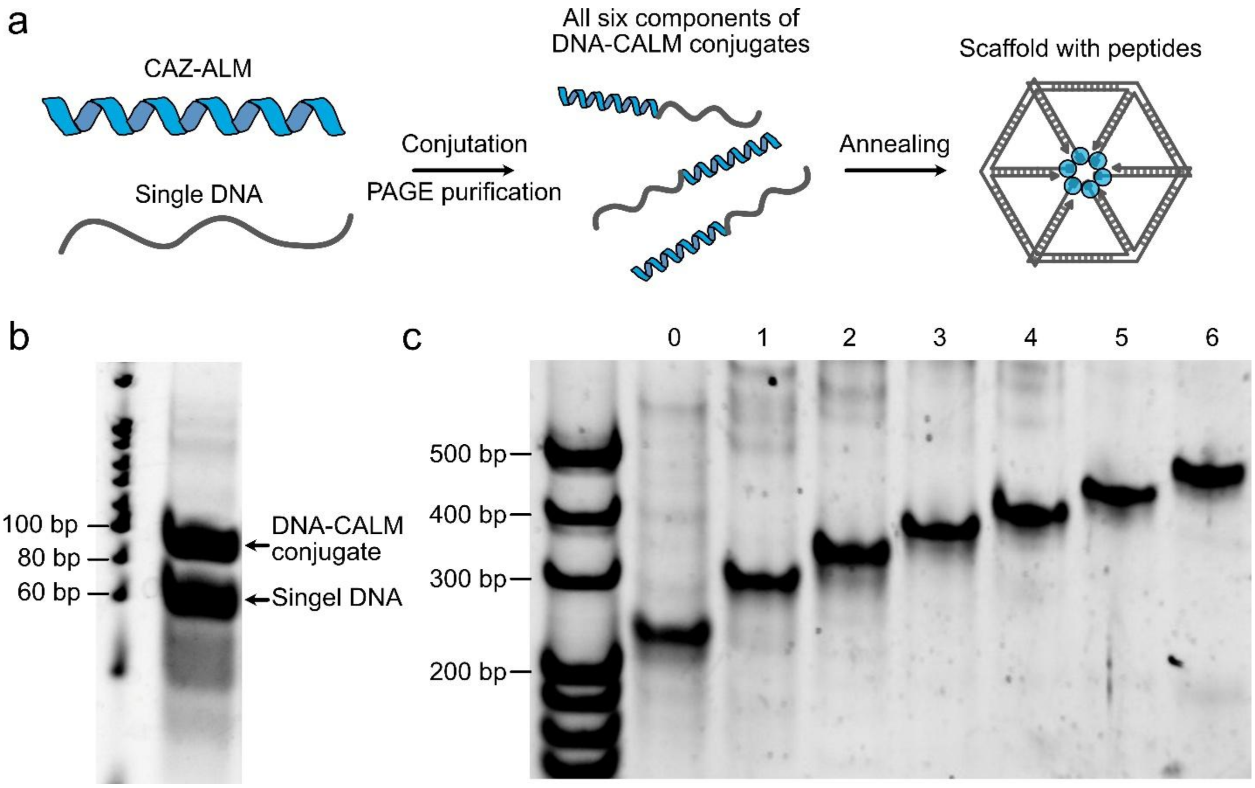

Figure S6. Verification of peptide numbers using the conjugation-first, assembly-later approach. (a) Schematic illustration of the approach. (b) 10% denaturing PAGE of the DNA and CALM peptide mixture. DNA–CALM conjugates were further extracted from the gel. Sf-1 DNA is shown as an example. (c) 5% SDS–PAGE of scaffolds containing different numbers of CALM peptides. The peptide numbers are indicated above the gel.

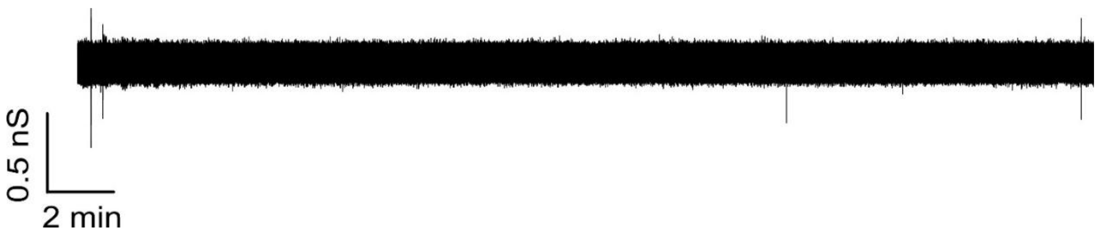

Figure S7. Prolonged electrical recordings were performed in the presence of 80 μM DDM at +150 mV. No apparent pore-formation signals were observed.

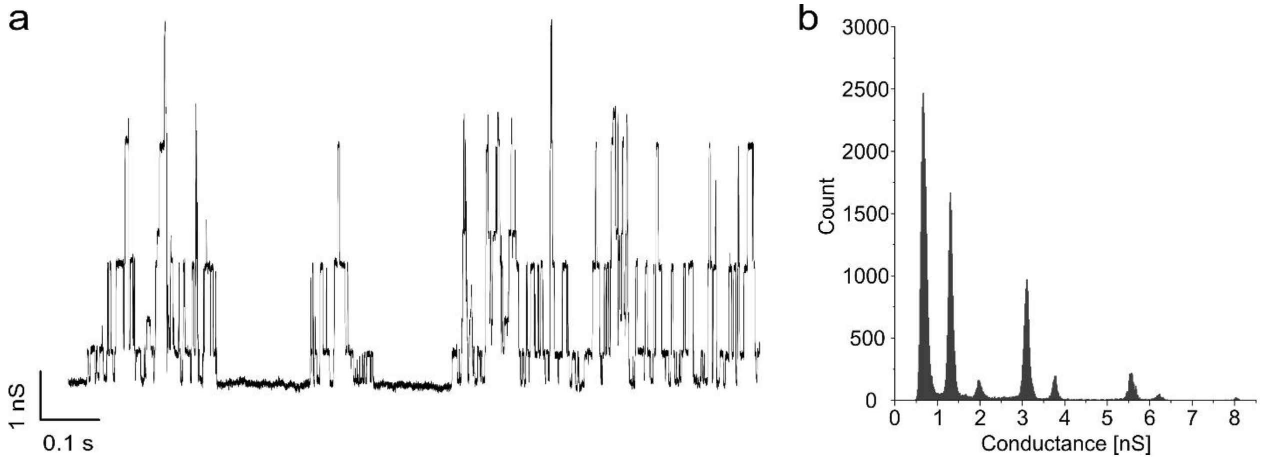

Figure S8. Electrical recordings of 500 nM CAZ-ALM at +200 mV. (a) Representative current trace and (b) conductance histogram.

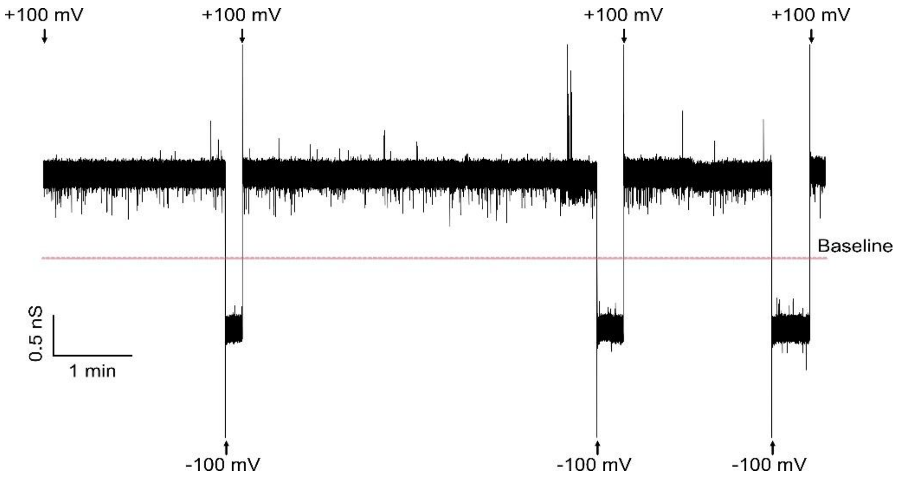

Figure S9. Prolonged electrical recordings of a single hexa-CALM nanopore. The pore was stable, and no clear gating was observed.

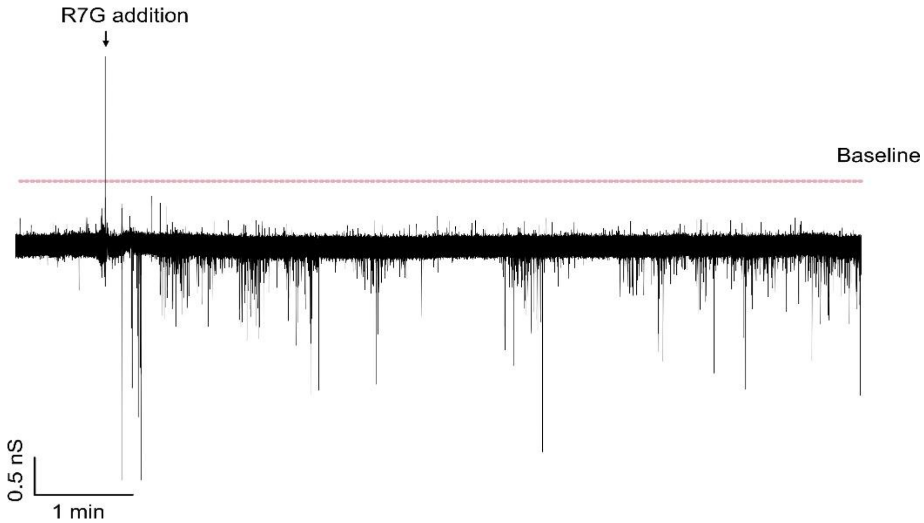

Figure S10. Single-molecule sensing experiments of R7G peptides using a single hexa-CALM nanopore at -100 mV. R7G peptide was added to the cis side of the chamber at a final concentration of 4 μM. After the addition of R7G, the pore became unstable, but no clear blocking signals from R7G were observed.

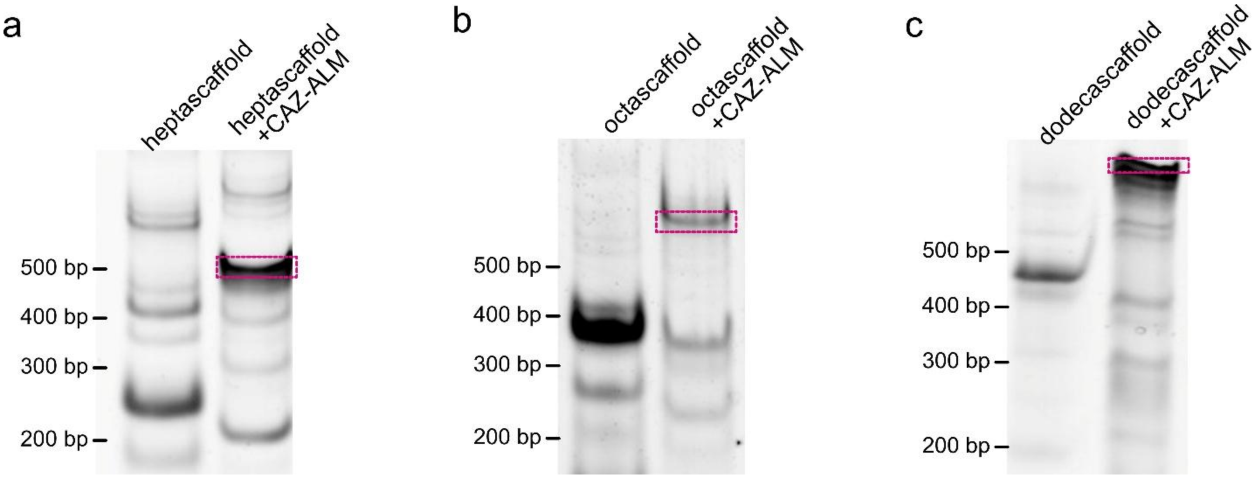

Figure S11. 5% PAGE analysis of (a) the heptascaffold, (b) the octascaffold, and (c) the dodecascaffold, together with their reaction mixtures with CAZ-ALM.

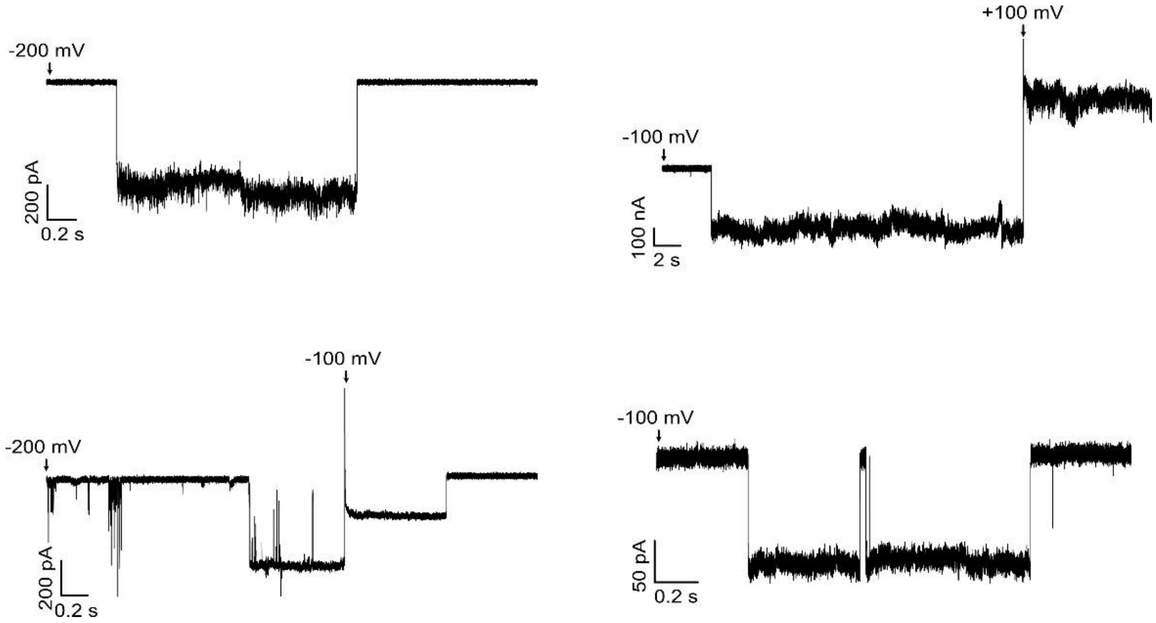

Figure S12. Other electrical recording signals from a single hepta-CALM nanopore. The pores were unstable and exhibited short lifetimes.

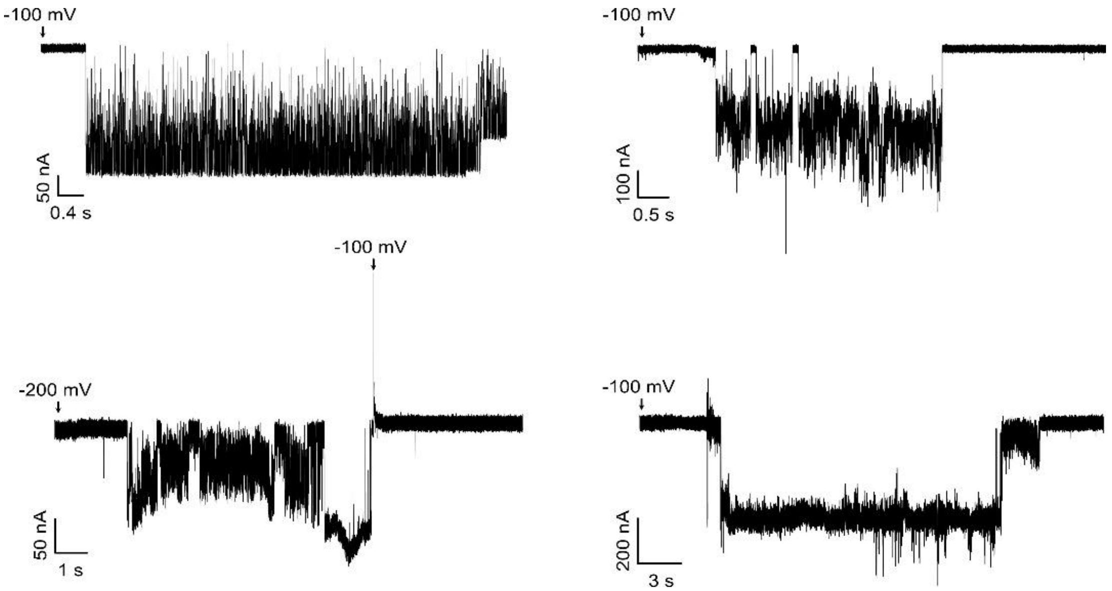

Figure S13. Other electrical recording signals from a single octa-CALM nanopore. The pores were unstable and exhibited short lifetimes.

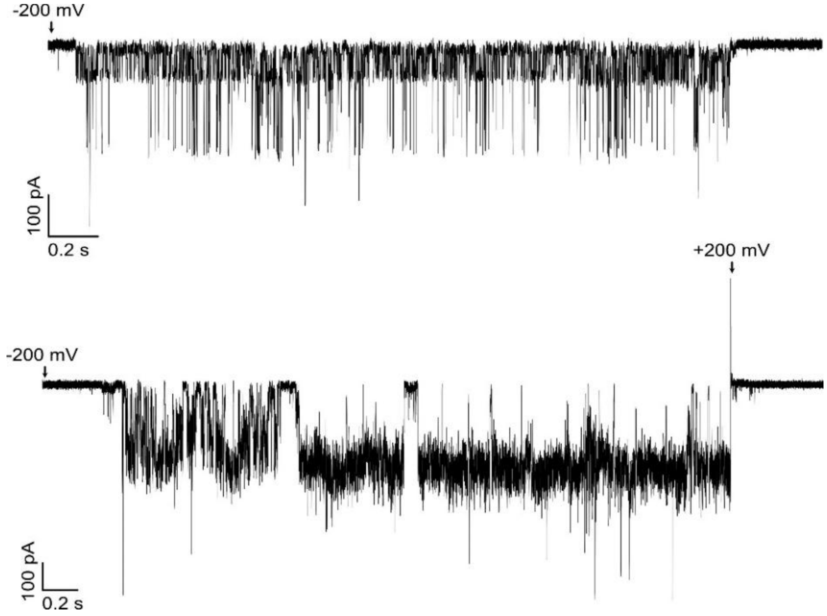

Figure S14. Other typical electrical recording signals from dodeca-CALM nanopores. Both multiple conductance states (top) and single pore insertions (bottom) were observed.

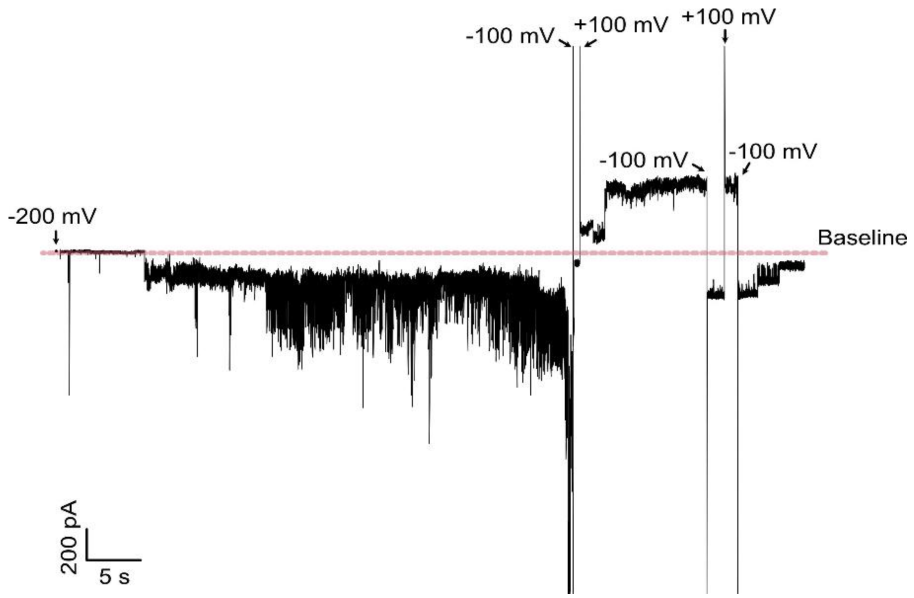

Figure S15. Another example of dodeca-CALM nanopores that were erratic at −200 mV but became stable at −100 mV.

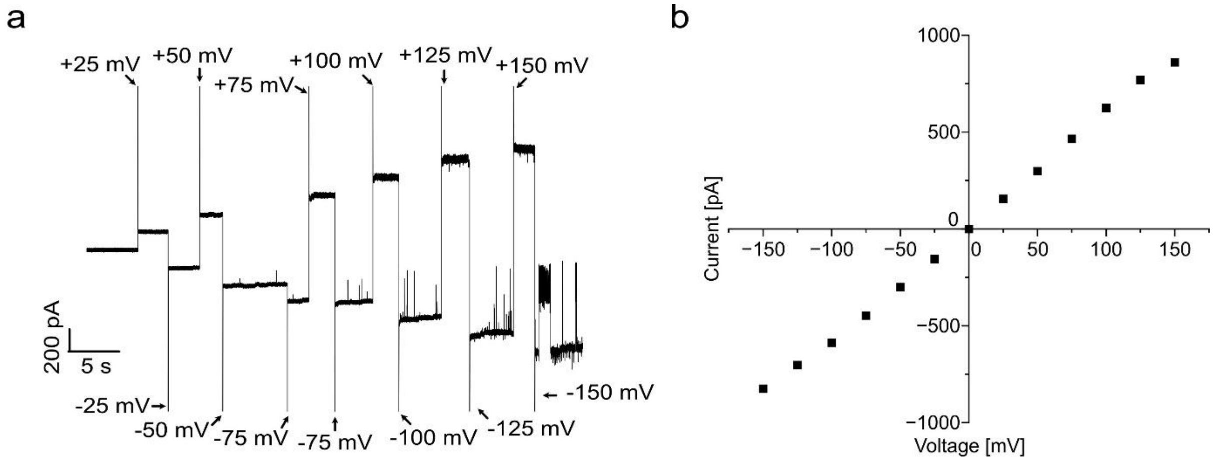

Figure S16. I-V (a) trace and (b) curve of the dodeca-CALM nanopore shown in Fig. 5(d).

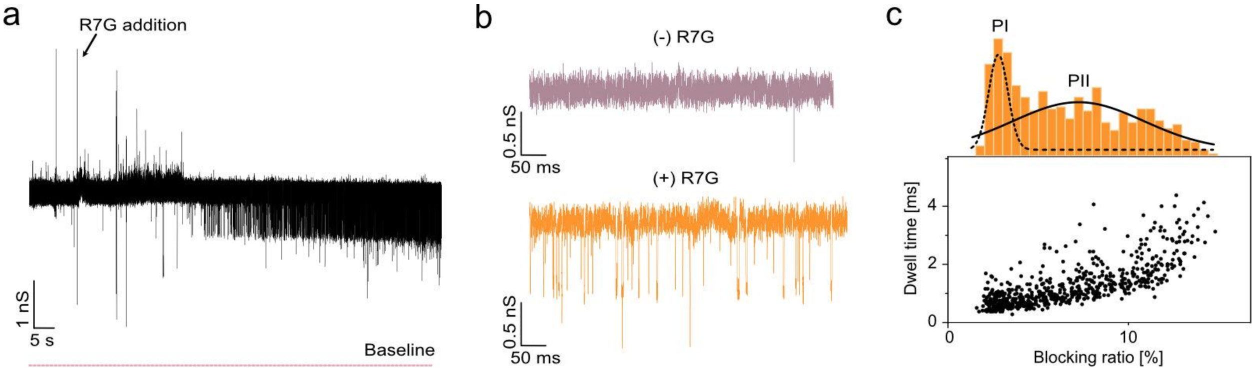

Figure S17. R7G peptide detection using the nanopore shown in Fig. 5g. (a) Current traces before and after the addition of R7G at a concentration of 40 μM. (b) Representative current traces of dodeca-CALM nanopores in the presence of 40 μM R7G, added to the trans chamber under an applied potential of +100 mV. (c) Scatter plot of dwell time versus blockade conductance for R7G signals recorded at +100 mV. The histogram shows the distribution of blocking ratios, fitted with a Gaussian function.

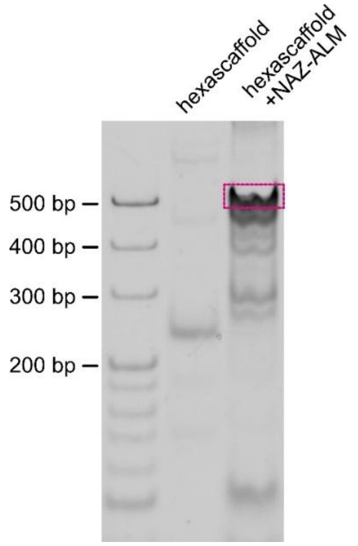

Figure S18. 5% PAGE of the reaction mixture of NAZ-ALM and the hexascaffold. The fully conjugated hexa-NALM was purified from the circled region of the gel.

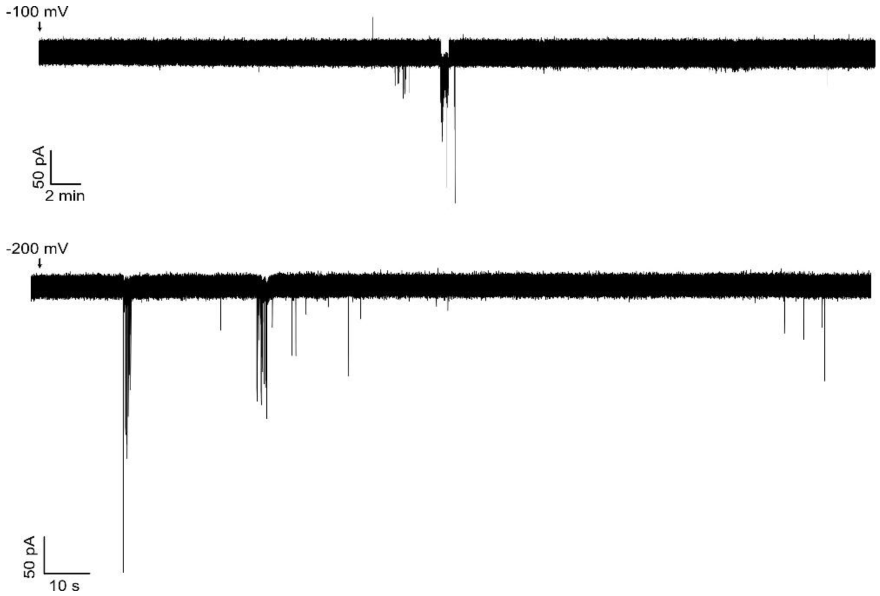

Figure S19. Typical electrical recording signals of hexa-NALM at −100 mV and −200 mV showed no clear step-like openings, but only bursts and spike-like events.

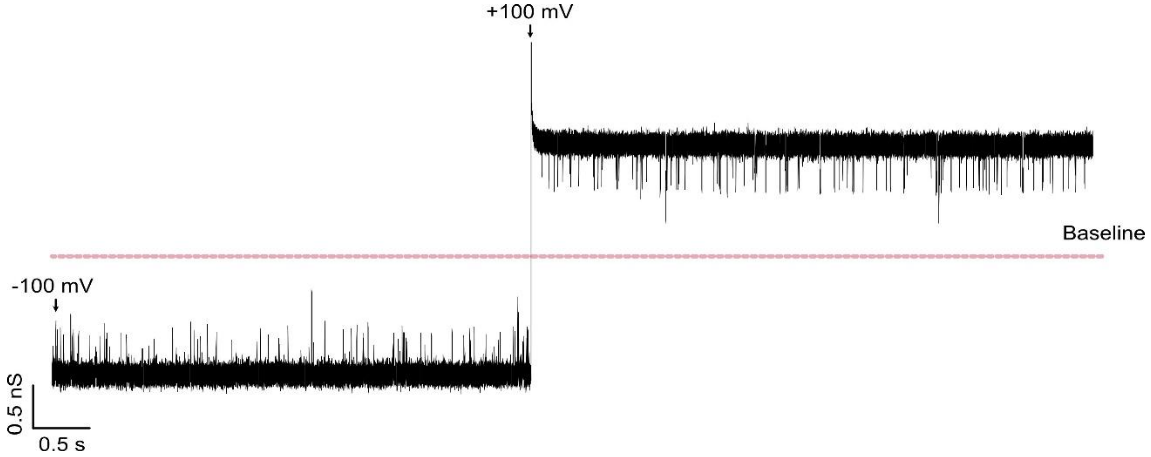

Figure S20. Gating behavior of hexa-NALM with conductance of around 1 nS. The current increased and decreased frequently.

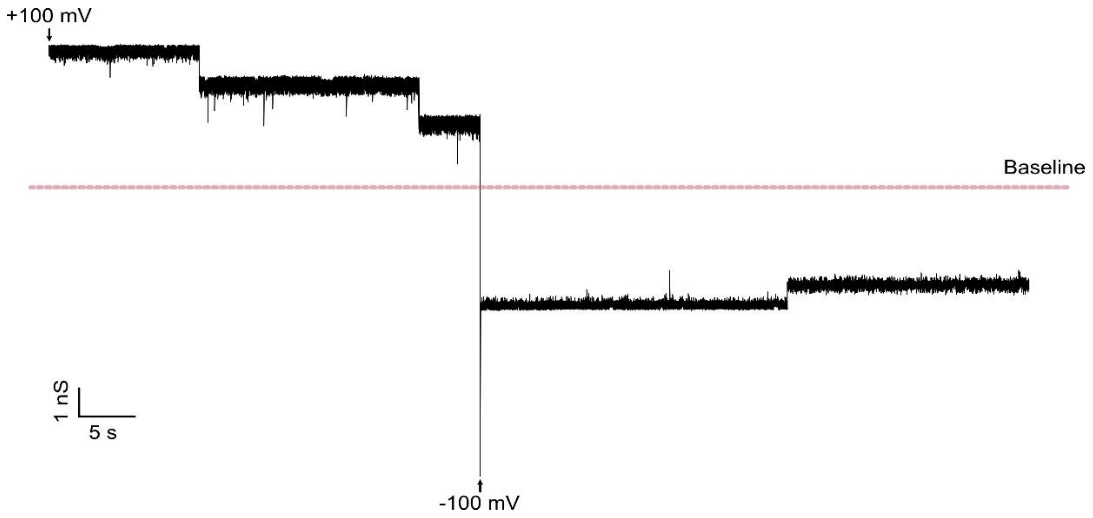

Figure S21. Gating behavior of hexa-NALM with conductance of 10 nS. The current dropped unilaterally and returned to the open level only when the applied potential was reversed.

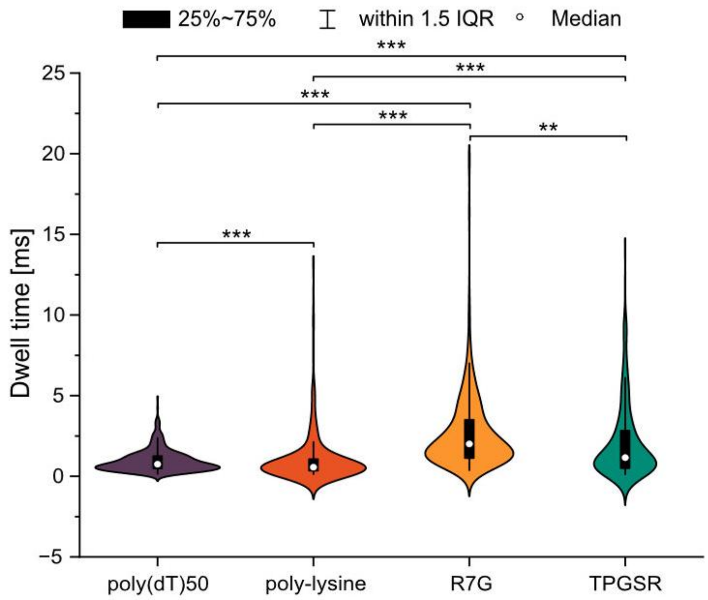

Figure S22. Violin plots of dwell times for translocation events of poly(dT)$_{50}$, PLL, R7G and TPGSR peptides through hexa-NALM nanopores. Statistical analysis using the Mann–Whitney U test confirmed significant differences between the datasets, with P < 0.01 and P < 0.001 indicated by ** and ***, respectively.

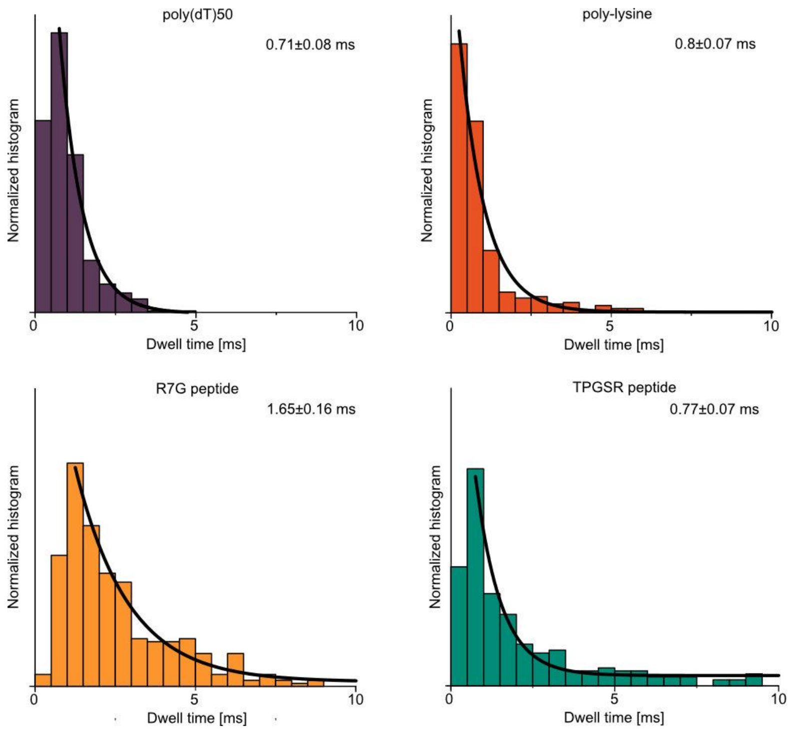

Figure S23. Histogram of dwell times for translocation events of poly(dT)$_{50}$, PLL, R7G and TPGSR peptides through hexa-NALM nanopores. Data were fitted to a single exponential decay function. The time constants are shown above.

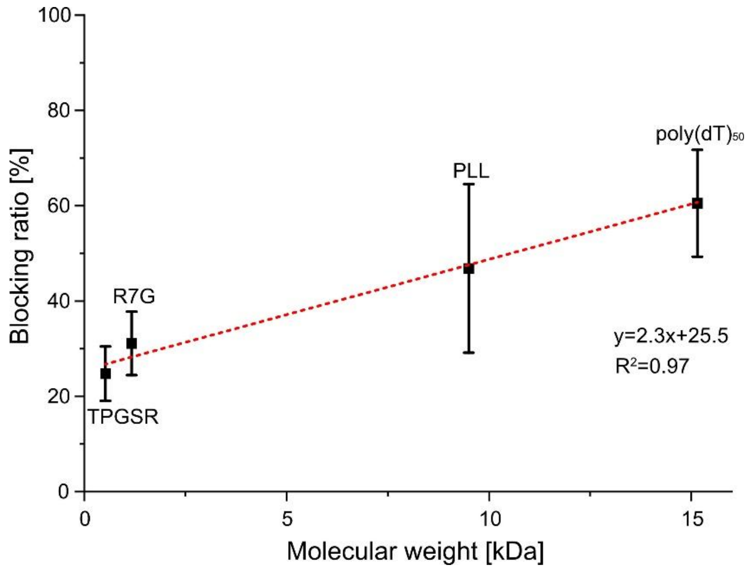

Figure S24. Average blocking ratio and its standard deviation produced by different sensing targets versus their molecular weight. More than 100 blocking events were used.

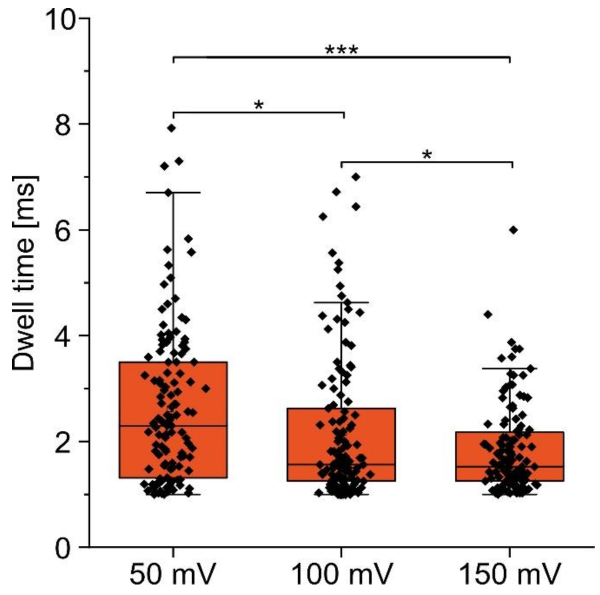

Figure S25. Dwell time of spike signals with durations > 1 ms. Error bars indicate SD. Statistical analysis using the Mann–Whitney U test revealed significant differences between datasets, with *P < 0.05 and ***P < 0.001, respectively. Data are derived from three independent experiments.

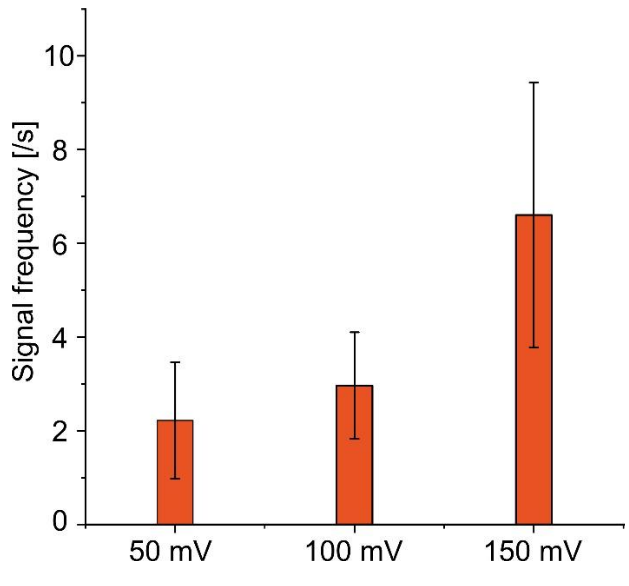

Figure S26. Signal frequency of PLL sensing at 2 μM in the trans side under applied potentials of +50 mV, +100 mV, and +150 mV. Error bars represent the standard deviation from three independent experiments.

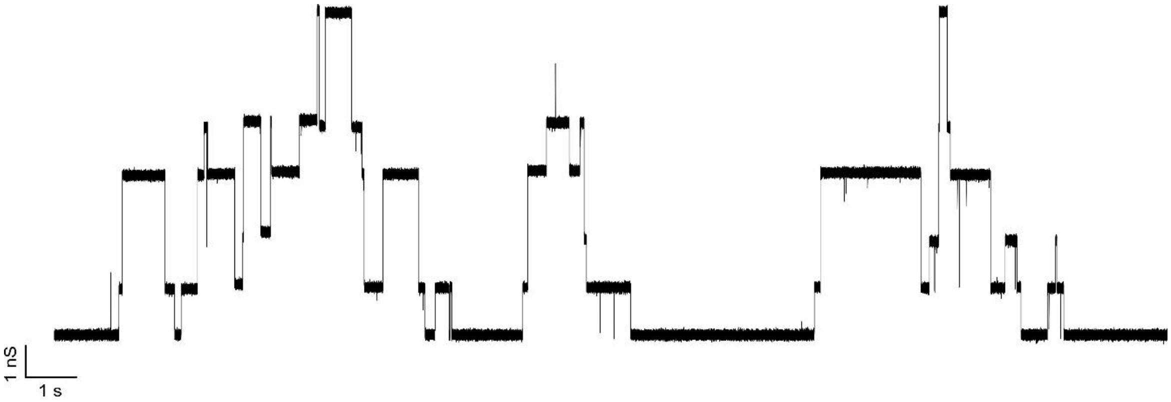

Figure S27. TPGSR peptide detection with di-ALM nanopores at a TPGSR concentration of 200 nM under an applied potential of 100 mV.

Supplementary explanations

Explanation S1. Estimation of current blockades

The current blockades arising from molecular translocation through the nanopore can be interpreted based on a geometric model. Assuming that the conductance scales with the available cross-sectional area, the relative blocking ratio, D, can be estimated as follows:[65]

$$D \simeq \frac{{d_{target}}^2}{d^2} \qquad (1)$$

where d and d_{target} is the effective diameter of nanopore and translocating molecule.

For the dodeca-CALM nanopore shown in Fig. 5g, an effective diameter of $d \approx 1.91$ nm was obtained using Hille's equation. Assuming an effective diameter of $d_{target} \approx 0.5$–0.8 nm for the R7G peptide, the expected blocking ratio is on the order of 7–17.5%. In our experiment, the blocking signals of R7G yielded two populations, labeled PI and PII, with peak blocking ratios of 3% and 7%, respectively (Fig. S17c). Based on previous studies of nanopore sensing using aerolysin nanopores,[86, 87] PI is attributed to collisions of R7G at the pore entrance, whereas PII is attributed to translocation of R7G through the dodeca-CALM nanopore, which is consistent with the estimated value.

For the hexa-NALM nanopore shown in Fig. 6f, with an effective diameter of 1.18 nm, the expected blocking ratio is on the order of 18–46% for peptides and approximately 70% for DNA ($d_{target} \approx 1$ nm). These estimated values are in reasonable agreement with the experimental blocking-ratio peaks of 21.8%, 32.5%, 36.5%, and 64.6% observed for TPGSR, R7G, poly(dT)$_{50}$, and PLL, respectively.

It should be noted that previous studies have suggested that volume exclusion alone is insufficient to describe changes in blocking ratios, as the solution conductivity inside the nanopore, which also contributes to the blocking ratio, changes upon target molecule entry.[71, 72] Therefore, the model presented above has certain limitations.

Explanation S2. Enlarging the pore size by leveraging helix dipole

When the DNA–scaffolded nanopores were added to the cis side of the chamber, we assumed that the peptide could only insert into the membrane from the terminus lacking the DNA scaffold. Under an applied potential, the transmembrane peptide portion of the DNA–scaffolded nanopores would experience a force at its cis end arising from a combination of helix-dipole–derived electrostatic force and a contact force that compensates for the electrostatic force acting on the DNA scaffold. Because the helix dipole is oriented from the negatively charged C-terminus to the positively charged N-terminus, with approximately −0.5 and +0.5 elementary charges at the two termini, respectively,[68], controlling the DNA attachment site allows us to regulate the direction of the electrical force exerted on the peptide. Meanwhile, DNA is a fully negatively charged polymer. As a result, in hexa-CALM, the dipole-driven electrostatic force and the DNA-derived interaction at the *cis* terminus act in the same direction, leading to a more stable configuration. In contrast, in hexa-NALM, these two forces act in opposite directions, creating a force imbalance. We infer that this imbalance may induce tilting of the ALM helices, which could in turn contribute to the formation of a larger pore.

Since the helix dipole is a common feature of helical peptides, this strategy is expected to be generally applicable. Nevertheless, it should be noted that pore formation behavior remains strongly dependent on the peptide sequence.
